# Pathway‐based stratification of gliomas uncovers four subtypes with different TME characteristics and prognosis

**DOI:** 10.1111/jcmm.18208

**Published:** 2024-04-13

**Authors:** Ruoyu Huang, Bo Han, Ying Zhang, Jingchen Yang, Kuanyu Wang, Xing Liu, Zhiliang Wang

**Affiliations:** ^1^ Department of Neurosurgery, Beijing Tiantan Hospital Capital Medical University Beijing China; ^2^ Department of Molecular Neuropathology Beijing Neurosurgical Institute, Capital Medical University Beijing China; ^3^ Department of Gamma Knife Center Beijing Neurosurgical Institute, Capital Medical University Beijing China

**Keywords:** gliomas, hypoxia, immune responses, metabolism, tumour microenvironment

## Abstract

Increasing evidences have found that the interactions between hypoxia, immune response and metabolism status in tumour microenvironment (TME) have clinical importance of predicting clinical outcomes and therapeutic efficacy. This study aimed to develop a reliable molecular stratification based on these key components of TME. The TCGA data set (training cohort) and two independent cohorts from CGGA database (validation cohort) were enrolled in this study. First, the enrichment score of 277 TME‐related signalling pathways was calculated by gene set variation analysis (GSVA). Then, consensus clustering identified four stable and reproducible subtypes (AFM, CSS, HIS and GLU) based on TME‐related signalling pathways, which were characterized by differences in hypoxia and immune responses, metabolism status, somatic alterations and clinical outcomes. Among the four subtypes, HIS subtype had features of immunosuppression, oxygen deprivation and active energy metabolism, resulting in a worst prognosis. Thus, for better clinical application of this acquired stratification, we constructed a risk signature by using the LASSO regression model to identify patients in HIS subtype accurately. We found that the risk signature could accurately screen out the patients in HIS subtype and had important reference value for individualized treatment of glioma patients. In brief, the definition of the TME‐related subtypes was a valuable tool for risk stratification in gliomas. It might serve as a reliable prognostic classifier and provide rational design of individualized treatment, and follow‐up scheduling for patients with gliomas.

## 1 INTRODUCTION

Gliomas are the most common and malignant type of primary brain tumour in adults with high mortality.^1^ Despite the combination of surgical resection and postoperative adjuvant radiotherapy and/or chemotherapy, the clinical outcomes in glioma patients are still unsatisfied.^2^ Meanwhile, the gliomas are rather resistant to targeted therapy and immunotherapy as well.^3^, ^4^ Gliomas evolve in complex tissue microenvironment, which they rely on for initiation, progression and metastasis. However, specifically blocking the pro‐tumorigenic tumour microenvironment (TME) is challenging undertaking.

In recent years, an increasing number of studies have revealed that bidirectional communication between cancer cells and tumour microenvironment is critical for tumour progression and patient prognosis, suggesting that remodelling of TME may be an effective strategy for treating cancer.^5^, ^6^ For example, oxygen deprivation, low pH, high concentration of lactate and peroxides have been reported as the characteristic hallmarks of solid cancers that directly contribute to tumour proliferation, invasion, metastasis and drug resistance.^7^, ^8^, ^9^, ^10^, ^11^ As an essential component of TME, immune cells infiltrating and residing in tumour tissues, including tumour‐associated macrophages (TAMs), T cells and dendritic cells (DCs) have complicated intercellular communication with cancer cells, which has a significant impact on metabolic state and biological functions of both sides.^12^, ^13^, ^14^ Interestingly, increasing evidence suggest that hypoxia was involved in regulating the metabolic state and biological functions of immune cells and cancer cells and the interaction between them in TME, although the underlying mechanisms remain unclear.^15^ Recently, we are gaining awareness that tumour cells can evade immune surveillance and anti‐tumour therapies via the functions of hypoxia and cell metabolism. Thus, the changing of hypoxic, immune and metabolism tumour microenvironment are emerged as essential targets for glioma.

The transcriptomics has emerged as a powerful manner for classification patients into different molecular subtype with distinct clinical outcomes. In 2010, The Cancer Genome Atlas (TCGA) Research Network performed high‐dimensional genomic classification and identified four molecular for GBM patients, referred to as classical (CL), mesenchymal (ME), neural (NE) and proneural (PN).^16^ However, the transcriptomic classification has failed to indicate the prognosis, temozolomide sensitivity and immunotherapy susceptibility for glioma. It is urgent to uncover the hypoxia, immune and metabolism‐based classifications and identify TME‐related subtypes in gliomas, which will facilitate the development of glioma treatments.

In this study, computational algorithms and transcriptomic data were employed to construct a valuable stratification for gliomas patients. We performed pathway‐based subtyping on glioma samples from Chinese Glioma Genome Atlas (CGGA) and TCGA data sets with 277 TME‐related signalling pathways, and the enrichment score of each pathway was calculated by gene set variation analysis (GSVA).^17^ We found that glioma samples from different data sets could be consistently divided into four robust subtypes with distinct biological features and clinical prognosis. Among them, HIS subtype had features of oxygen deprivation and immunosuppression, leads to the worst prognosis of patients. The single cell RNA sequencing (scRNA‐seq) analysis indicated that HIS subtype cells had a mesenchymal‐like phenotype and become more malignant by interacting with immune cells. To improve the clinical application value of this pathway‐based subtyping, we also constructed a risk signature by LASSO COX regression model to identify patients in HIS subtype. The survival analysis suggested that patient in high‐risk group was more sensitive to combined chemo‐radiotherapy and PD‐1 targeted therapy. Our findings proposed a new insight into the classification of gliomas and had important reference value for prognosis prediction and individualized treatment of gliomas.

## 2 MATERIALS AND METHODS

### 2.1 Patients and data sets

A total of 1978 glioma samples were enrolled in this study. The Cancer Genome Atlas (TCGA) mRNA sequencing data set, which contains a total of 960 glioma samples was downloaded from public databases (https://portal.gdc.cancer.gov/) and used as discovery cohort.^18^ The Chinese Glioma Genome Atlas (CGGA) data set1 and data set2 (including 693 and 325 samples, respectively) were served as independent validation cohorts. In CGGA data sets, the transcriptome sequencing data and clinical information were downloaded from CGGA databases (http://www.cgga.org.cn). The single‐cell RNA sequencing data were also obtained from CGGA databases. The details of establishing and managing CGGA databases have been mentioned in our previous studies.^19^ This study was approved by Institutional Review Board (IRB) of Beijing Tiantan Hospital Affiliated to Capital Medical University, and wrote informed consent was obtained from each patient. GSE16011 data set was enrolled in this study to further validated the prognostic value of the pathway‐based subtypes.^20^ The cohort deposited in SRA PRJNA482620 was enrolled in this study to investigate the association between risk signature and response to PD1 inhibitor treatment.^21^ Besides, the GSE121810 data set was employed to find an optimal immunotherapy strategy for patients with gliomas.^22^

### 2.2 Identification of TME‐related subtypes of gliomas

The enrichment score of TME‐related signalling pathways were calculated by R package ‘GSVA’. The median absolute deviation (MAD) was used to assessed the underlying variation among patients for enrichment score of all signalling pathways. Unsupervised clustering was performed using the R package ‘ConsensusClusterPlus’ for cluster discovery based on the activation of hypoxia response, metabolism and immune‐related signalling pathways.^23^ The optimal K was selected by cumulative distribution function (CDF) curves and consensus heatmap.

### 2.3 Construction of the risk signature

The R package ‘limma’ was applied to identify differentially expressed genes among four clusters.^24^ Then, the genes with the highest expression level in Cluster3 were selected to construct a Cluster3‐related risk signature. Subsequently, the least absolute shrinkage and selection operator (LASSO) analysis, which was suitable for high‐dimensional regression analysis, was employed to identify the most valuable predictive genes to construct an optimal signature with R package ‘glmnet’.^25^ The risk score for each patient was calculated as following formula: $\text{Risk score}=\sum_{i=1}^{n}\text{βixi}$. In this formula, βi was the regression coefficient obtained from LASSO Cox analysis and xi was the relative expression level of each selected genes.

### 2.4 Immunohistochemical staining

A total of 20 paraffin‐embedded glioma tissues from CGGA database were collected for immunohistochemical (IHC) analysis. Anti‐CA9 [1:500, 11071‐1‐AP, Proteintech (Wuhan, Hubei, PR China)], C20orf20 (1:500, 26040‐1‐AP, Proteintech) and anti‐FOSL1 [1:500, A5372, ABclonal (Wuhan, Hubei, PR China)] were used to evaluate the hypoxia response of glioma samples. Anti‐CD163 [1:500, ab182422, Abcam (Burlingame, CA, USA)] was used to detect M2 macrophages. Anti‐CD4 (1:500, ab133616, Abcam) was used to detect CD4+ T cells. Anti‐PKM2 [1:500, 15822‐1‐AP, Proteintech (Wuhan, Hubei, PR China)] and anti‐DPYD (1:500, 27662‐1‐AP, Proteintech) were used to estimate glucose metabolism and pyrimidine metabolism of glioma samples, respectively. Briefly, after deparaffinizing, the four‐micrometre‐thick sections were boiled with EDTA antigen retrieval buffer. Then, the sections were incubated with primary antibodies overnight at 4°C. After that, the sections were incubated with propriate secondary antibody at room temperature for 1 h. Finally, the sections were stained with DAB (ZSGB‐BIO, Beijing, P.R. China) and haematoxylin (Solarbio, Beijing, PR China). Finally, place the tissue sections under a microscope with a magnification of 20× for observation and capturing images. The stained slides were individually evaluated by two experienced pathologists.

### 2.5 Bioinformatic analysis

Graph learning‐based dimensionality reduction analyses were performed to further explore the intrinsic distribution of individual patients. The discriminative dimensionality reduction with trees (DDRTree) was used to visualize the TME landscape, and the plot cell trajectory function was calculated by monocle with R package ‘monocle’.^26^, ^27^ Cases with somatic mutations and somatic copy number alternations (CNAs) were downloaded from TCGA database to investigate the differences of genomic alterations among four clusters. The CNAs were analysed by GISTIC 2.0.^28^ The stromal score and immune score, which represents the degree of stromal and immune cells infiltration in glioma samples were calculated by ESTIMATE algorithm with R package ‘estimate’.^29^ The tumour purity of each sample was obtained based on the methods proposed by Yoshihara K et al.^29^ Principal components analysis (PCA) was applied to detect the differences of transcriptome profiles among groups.^30^ Gene ontology (GO) analysis and gene set enrichment analysis (GSEA) were carried out for functional annotation.^31^, ^32^ Receiver operating characteristic (ROC) curve analysis was performed to predict patients from specific cluster, and timeROC curve analysis was performed for 1‐, 3‐ and 5‐year overall survival (OS) prediction.^33^, ^34^ The R package ‘pROC’ and ‘timeROC’ was used in ROC and timeROC curve analysis, respectively.

The scRNA‐seq data were analysed by R package ‘Seurat’. The CellPhoneDB software was used to identified the significant ligand‐receptor interactions between tumour cell and immune cells.

### 2.6 Statistical analysis

In this study, Student's *t*‐test and one‐way ANOVA were conducted to estimate statistical differences among groups. Pearson's correlation analysis was performed to detect the correlation between two continuous variables. The Kaplan–Meier (K–M) survival curves analysis with long‐rank test was performed to investigate survival distributions of stratified patients. Cox regression analysis was used to evaluate the independent prognostic value of the risk signature. The statistical analyses were mainly conducted using R language (version 3.6.2) and SPSS (version 16.0). *p* < 0.05 was regarded as statistically significant.

## 3 RESULTS

### 3.1 Consensus clustering identifies four subtypes of gliomas

**FIGURE 1:**
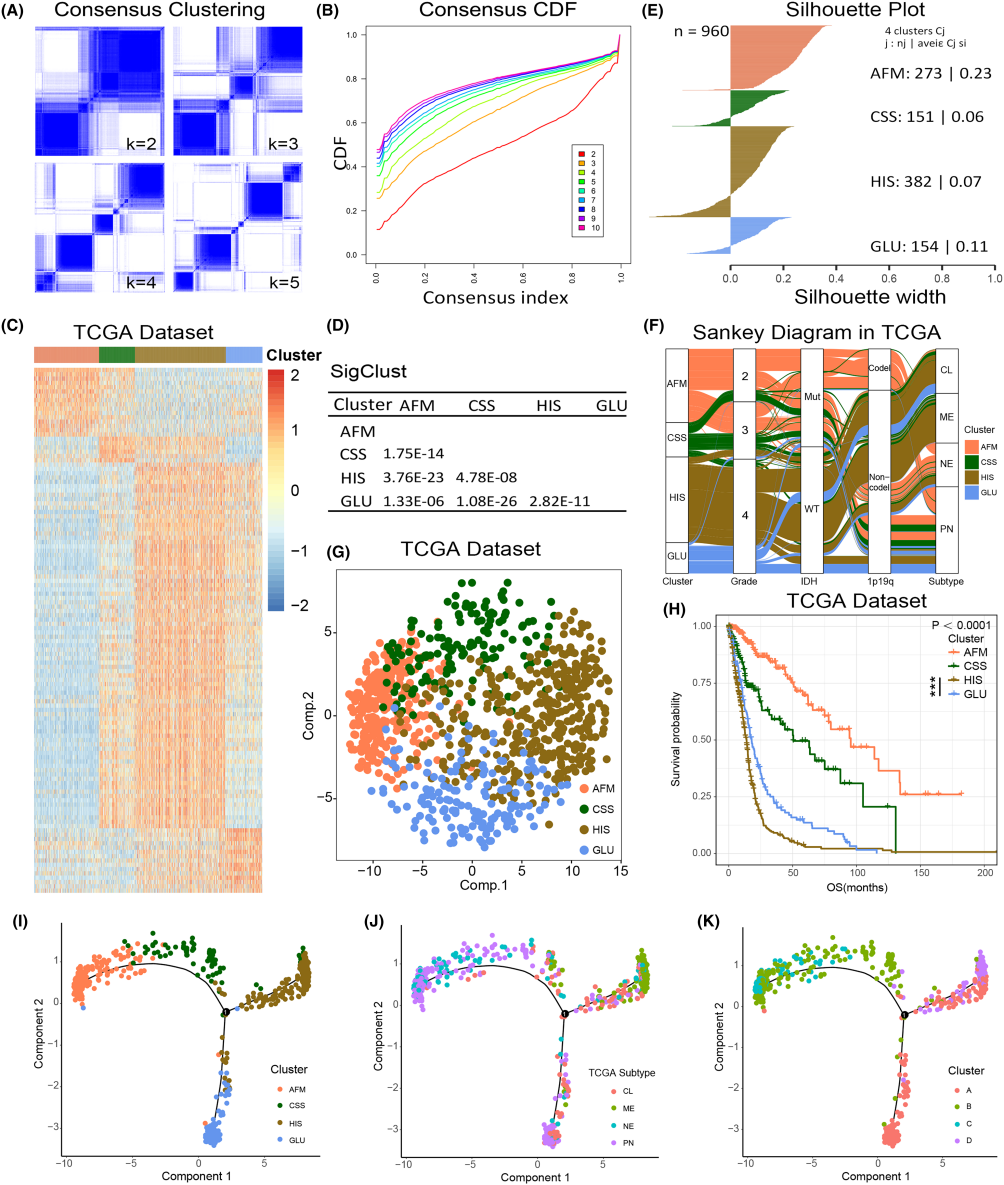
Identification of four TME‐related subtypes in gliomas in TCGA data set. (A) Consensus clustering matrix of 962 TCGA samples for *k* = 2 to *k* = 5. (B) Consensus clustering CDF for *k* = 2 to *k* = 10. (C) Heatmap of four subtypes defined by TME‐related signalling pathways in TCGA data set. (D) SigClust p values for all pairwise comparisons of clusters. (E) Silhouette plot for identification of core samples. (F) Sankey diagram exhibiting the association between cluster and clinicopathological characteristics of glioma samples in TCGA data set. (G) PCA analysis of four subtypes in TCGA data set. (H) Kaplan–Meier analysis of four clusters based on overall survival (OS) in TCGA data set. (I–K) Graph learning based dimensionality reduction analysis to the TME landscape in gliomas. Each point represents a patient with colours corresponding TME subtypes (I), TCGA molecular subtypes (J), and DDRTree subtypes (K).

Hypoxia, immune responses, and cell metabolism were key components of TME of gliomas. In order to stratify the glioma patients by the differences of TME characteristics, 277 related signalling pathways were obtained from MSigDB (Supplementary Figure S1A,B, Supplementary Table S1) and our approach and workflow for this study were summarized in Supplementary Figure S1C. The enrichment score of each pathway was calculated by GSVA. After that, consensus clustering analysis of 166 pathways with highly variable enrichment score (MAD >1) in TCGA RNA sequencing data set identified four main subtypes, the optimal number of clusters (*k* = 4) was assessed by cumulative distribution function (CDF) curves and consensus matrices (Figure 1A–C). Then, we preformed SigClust analysis to evaluate the cluster significance and found that all cluster boundaries were statistically significant (Figure 1D). The pathways most representative of the subtypes were identified based on their positive silhouette width, indicating higher similarity to their own class than to any other class member (Figure 1E). Consistent subtypes were also obtained in two independent CGGA RNA sequencing and GSE16011 data sets (Supplementary Figure S2A–C). According to the core pathways of each subtype (Supplementary Table S2) and the functional analysis we mentioned blow, C1 subtype was defined as amino/fatty acid metabolism (AFM, marked in red throughout the manuscript, respectively), C2 subtype was defined as cytokine secretion suppression (CSS, green). C3 subtype was defined as hypoxia/immunosuppression (HIS, brown), and C4 subtype was defined as glucose metabolism (GLU, blue).

In TCGA data set, the Sankey diagram showed significant differences in clinicopathological characteristics among four subtypes (Figure 1F). PCA was performed based on the enrichment score of signalling pathways in TCGA data set. The results showed that cases in four clusters tended to distribute in different directions. (Figure 1G). Besides, the K‐M survival analysis suggested that the group membership of each cluster was associated with distinct survival characteristics (Figure 1H). Among them, AFM and CSS subtypes tended to have a better clinical outcome, while the prognosis of HIS and GLU subtypes was poor (Figure 1H). Furthermore, between the HIS and GLU subtypes, patients with the HIS subtype exhibit a worse prognosis (Figure 1H). The reproducibility of these findings was also assessed in CGGA and GSE16011 data sets (Supplementary Figure S2F–K).

To explore the TME landscape of gliomas, we further applied the graph learning‐based dimensionality reduction analysis to the enrichment score of TME‐related signalling pathways.^26^, ^27^ We found that the integral distribution of AFM subtype was opposite to that of HIS and GLU subtypes (Figure 1I). Meanwhile, AFM and CSS subtypes were distributed in the same direction, suggesting these two subtypes may have similar TME characteristics. Besides, the results also revealed that compared with TCGA subtypes and DDRTree subtypes, our pathway‐based classification could more efficiently stratify gliomas patients with TME characteristics (Figure 1I–K). By performing ROC curve analyses, we also found that the TME pathway‐based subtypes had the highest prognostic value compared with TCGA subtypes and DDRTree subtypes (Supplementary Figure S2I).

### 3.2 The subtypes associated with distinct patterns of genomic alterations

**FIGURE 2:**
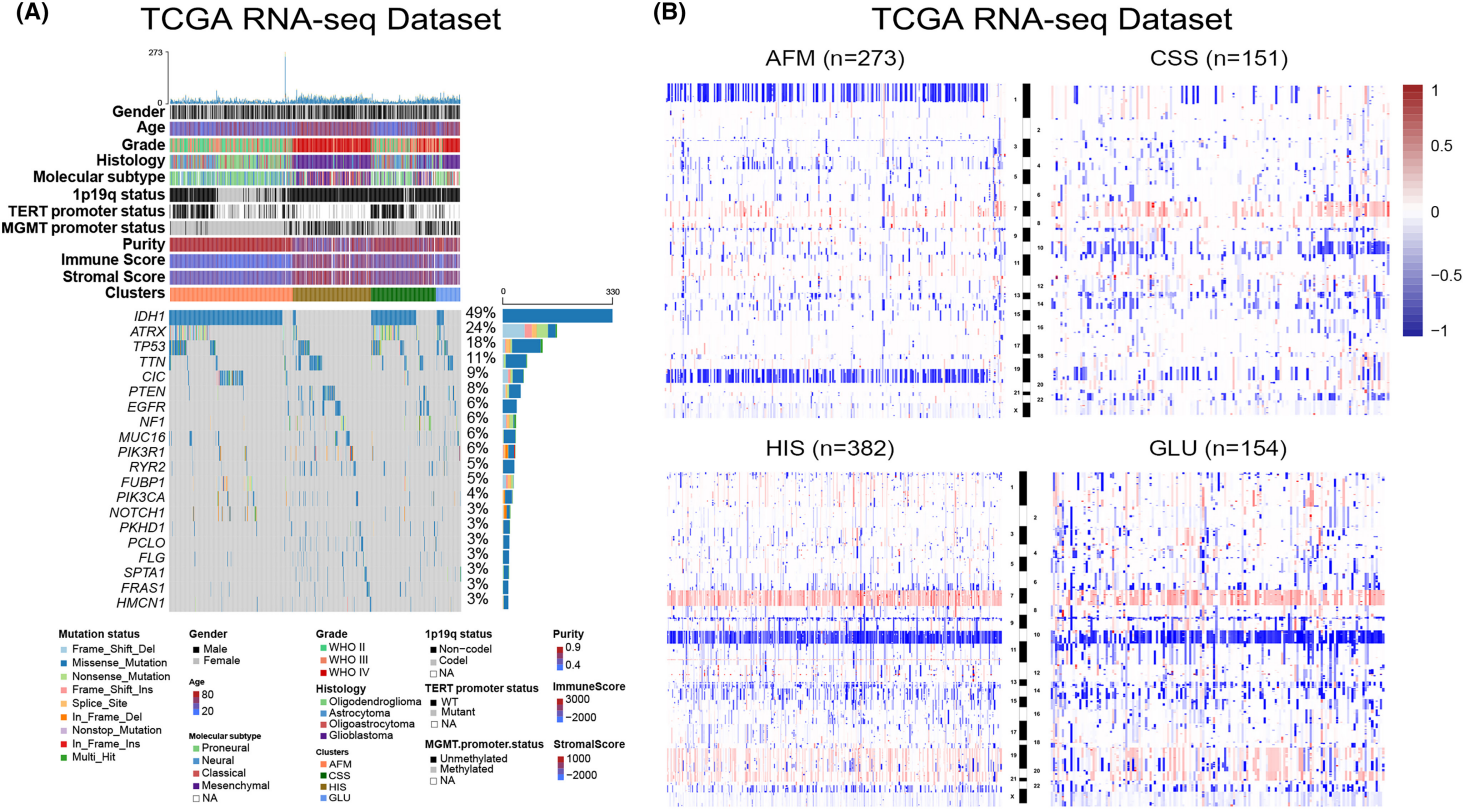
Comparison of genomic alterations among four subtypes in the TCGA data set. (A) Somatic mutation analysis among four subtypes. (B) Distinct CNA profiles among four subtypes.

Somatic mutations and copy number alterations (CNAs) have significant impact on the biological characteristics and clinical prognosis of gliomas.^35^, ^36^ Thus, samples with somatic mutations and CNAs information in TCGA data set were enrolled in this study to further explore the molecular mechanisms influencing the hypoxia response, immune response, and metabolic regulation of gliomas. After comparing the frequency of mutations among samples in four subtypes, we found that more somatic mutations were revealed in samples from HIS and GLU subtypes (Figure 2A). However, the *IDH1* mutation and *ATRX* mutation tended to be enriched in AFM and CSS subtypes (Figure 2A). Besides, The *CIC* mutation and *FUBP1* mutation were significantly enriched in AFM subtype (Supplementary Figure S3A). A higher incidence of *TTN* mutation was observed in both CSS, HIS and GLU subtypes (Supplementary Figure S3B–D). Meanwhile, the results also showed that the cases from HIS subtype had the highest *PTEN* mutation rate and the lowest *TP53* mutation rate (Supplementary Figure S3C). Furthermore, the CNAs among cases in four clusters were also investigated in TCGA data set. We found that the 1p/19q codeletion, as a representative genomic alteration in oligodendroglioma was mainly observed in cases from AFM (Figure 2B). However, chromosome 7 amplification accompanied chromosome 10 loss, which were usually considered as a genomic hallmark of GBM tended to be enriched in HIS and GLU subtypes (Figure 2B). For cases from CSS, the CNAs were insignificant, suggesting CNAs may not play an essential role in this subtype of gliomas. These findings revealed that cases in four subtypes were inconsistent on genomic alterations, which may provide new insights into the hypoxia response, immune response and metabolic regulation of gliomas.

### 3.3 The four subtypes differ in tumour immunologic microenvironment

**FIGURE 3:**
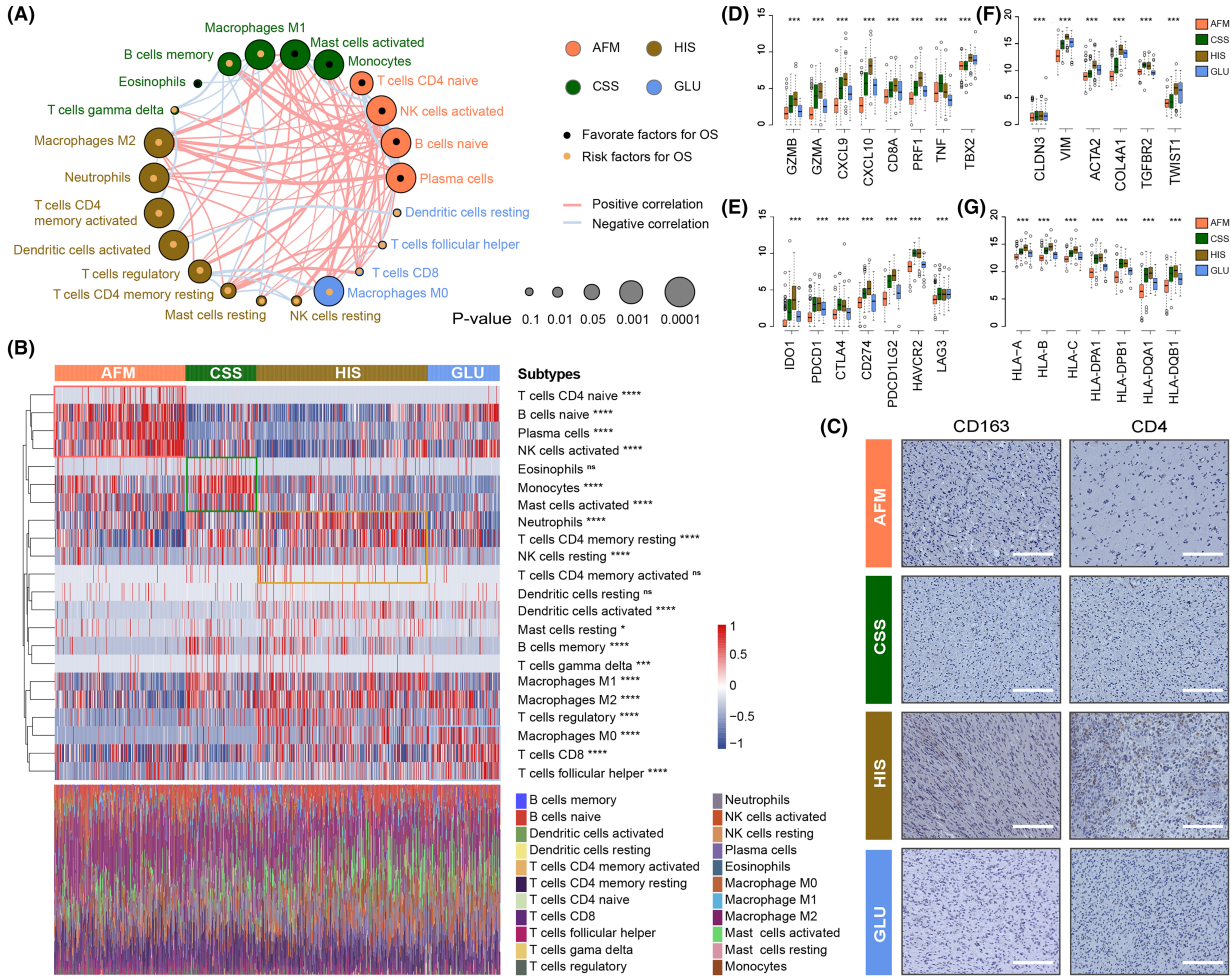
Landscape of the immune responses in gliomas and characteristics of four subtypes. (A) The intercellular interaction of various cell types in TME and their correlation with four subtypes. The strength of cellular interactions was estimated by Spearman correlation analysis. (B) Unsupervised clustering of TME cells for 962 patients in the TCGA data set. Four subtypes, immune score, stromal score, tumour purity and EMT score were shown as patient annotations. (C) IHC analysis of CD4 and CD163 in four subtypes. (D) The expression level of immune activation‐relevant genes (*GZMB*, *GZMA*, *CXCL9*, *CXCL10*, *CD8A*, *PRF1*, *TNF*, and *TBX2*) in four TME‐related subtypes. (E) The expression level of immune checkpoints (*IDO1*, *PDCD1*, *CTLA4*, *CD274*, *PDCD1LG2*, *HAVCR2*, and *LAG3*) in four TME‐related subtypes. (F) The expression level of HLA complex‐relevant genes (*HLA‐A*, *HLA‐B*, *HLA‐C*, *HLA‐DPA1*, *HLA‐DPB1*, *HLA‐DQA1*, and *HLA‐DQB1*) in four TME‐related subtypes. (G) The expression level of TGF‐β/EMT pathway‐relevant genes (*CLDN3*, *VIM*, *ACTA2*, *COL4A1*, *TGFBR2*, and *TWIST1*) in four TME‐related subtypes. *p < 0.05, **p < 0.01, ***p < 0.001, ****p < 0.0001.

To explore the immune heterogeneity among the four subtypes, immune‐related signatures published recently were included in this study. First, the stromal and immune score calculated by Estimate algorithm was used to examine the distribution of stromal and immune content among four subtypes in TCGA data set (Supplementary Figures S3B and S4A).^29^ We found that HIS had the highest stromal and immune scores among the four subtypes (Supplementary Figure S4A,B). On the contrary, we observed a significant reduction in tumour purity of samples in HIS subtype (Supplementary Figure S4C), suggesting that tumours in HIS subtype contained larger number of immune cells (Supplementary Figure S4C). We also validated these findings in CGGA data sets and similar results were obtained (Supplementary Figure S4D–I). Besides, a tumour microenvironment (TME) cell network was constructed to depict an overview of immune cell interactions and their effects on the clinical outcome of patients with gliomas (Figure 3A). The NK cells activation and the enrichment of plasma cells, naïve CD4+ T cell and B cell may be the main reasons for the better prognosis of patients in AFM subtype (Figure 3A,B). For patients in HIS subtype, the M2 macrophage, neutrophils, activated DCs and activated memory CD4+ T cells may contribute to the poor prognosis (Figure 3A,B). CSS showed significant increases in the infiltration of monocytes, M1 macrophages, and activated mast cells, while GLU exhibited high infiltration of M0 macrophages, CD8+ T cells and follicular helper T cells (Figure 3A,B). To verify this, paraffin‐embedded glioma tissues from CGGA database were collected for IHC analysis, and the results showed that CD4 and CD163 were overexpressed in glioma tissues of HIS subtype (Figure 3C). These findings suggested that there were significant differences in TME cell infiltration in the four subtypes of gliomas.

To further investigate the immune background characterizing each TME‐related subtype, we also analysed the expression level of selected cytokine, chemokine, and immune checkpoint in glioma samples from TCGA data set (Figure 3D,E). In this part, we considered *GZMB*, *GZMA, CXCL9, CXCL10, CD8A, PRF1, TNF* and *TBX2* to be immune‐activated biomarkers. As shown in Figure 3C, HIS subtype was associated with high expression of T helper 1 (Th1)/cytotoxic T lymphocyte (CTL)‐related genes (Especially *CXCL9*, *CXCL10*, *GZMA*, and *GZMB*)^37^ (Figure 3D). These results suggested that the HIS has the highest level of immunocyte infiltration. Additionally, we further investigated the expression of several immune checkpoint genes, since T cell and nature killer (NK) cell exhaustion has been identified as an important mechanism of cancer progression and immune evasion.^38^ We also found that the HIS subtype had the highest expression of several immune checkpoints (particularly *IDO1, CD274* and *PDCD1LG2*), it means that glioma samples from HIS subtype tend to be immunosuppressed (Figure 3E). Besides, the key members of human leukocyte antigen (HLA) complex, which are required to present endogenous cellular antigens to circulating T cells were also included in this study (Figure 3F). The results revealed that CSS and HIS subtypes were associated with high expression of HLA complex member mRNAs. Moreover, the expression level of TGF‐β/EMT pathway related genes (such as *VIM, ACTA2, COL4A1* and *TWIST1*) were relatively higher in HIS and GLU subtypes (Figure 3G). In brief, the results mentioned above identified an extensive immune heterogeneity among four subtypes of gliomas.

### 3.4 The clusters are associated with hypoxia and metabolic status in gliomas

**FIGURE 4:**
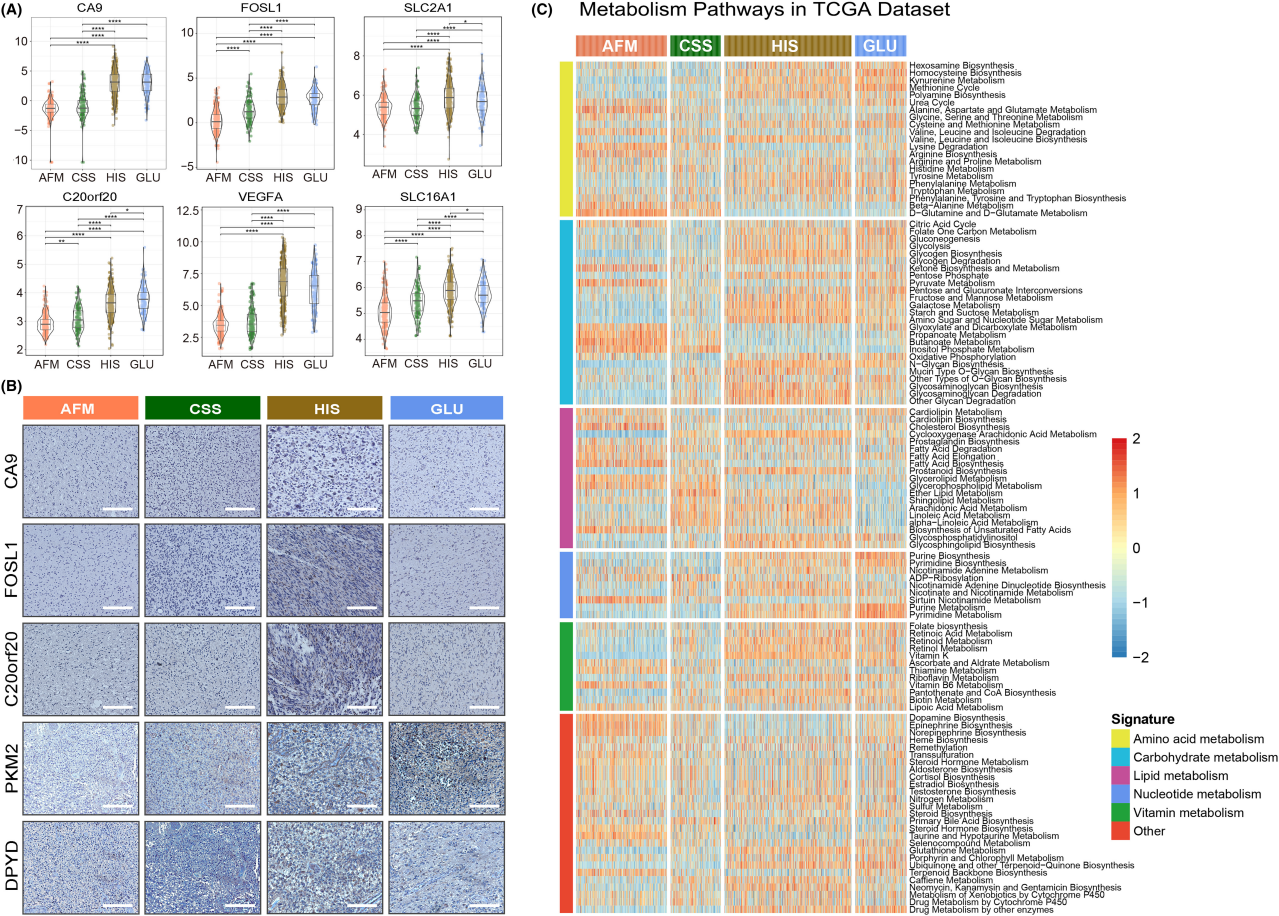
Associations of TME‐related subtypes with hypoxia and metabolism status in gliomas. (A) The expression level of biomarkers of hypoxia (CA9, FOSL1, SLC2A1, SLC16A1, C20orf20, and VEGFA) in four TME‐related subtypes in TCGA data set. (B) IHC analysis of biomarkers of hypoxia response and metabolic pathways in four subtypes. (C) The differences in the activation of cellular metabolic pathways among four subtypes in TCGA data set.

According to the previous researches, the hypoxia and metabolic status play an essential role in the malignant progression of human cancers.^7^, ^39^ The activation of anti‐tumour immune response is also affected by the oxygen content and cellular metabolism in tumour microenvironment. Thus, we further investigated the differences of hypoxia response and metabolic status among the four subtypes. In this study, *CA9, FOSL1, SLC2A1, SLC16A1, C20orf20* and *VEGFA* were considering as biomarkers of hypoxia response according to the related literature.^40^ After exploring the expression level of these hypoxia response‐related biomarkers in TCGA data set, we found that the glioma samples from HIS and GLU subtypes were in a more obvious hypoxia state (Figure 4A). Besides, IHC analysis was performed with paraffin‐embedded glioma tissues from CGGA database, and similar results were obtained (Figure 4B). Besides, we also found that the glioma samples from four clusters have significant differences in cellular metabolic pathways (Figure 4C, Supplementary Figure S5). Notably, in HIS and GLU subtypes with poor prognosis, the metabolism of nucleotides, glucose and vitamins was more active (Figure 4C, Supplementary Figure S5). In contrast, glioma samples with active amino acid and fatty acid metabolism tend to be enriched in AFM and CSS subtypes (Figure 4C, Supplementary Figure S5). The differences in metabolic pathways among the four clusters were also verified by IHC (Figure 4). These differences in hypoxia response and cellular metabolism may be the molecular mechanism behind the heterogeneity of tumour immunologic microenvironment, which also has a significant impact on the prognosis of patients with gliomas.

### 3.5 Validation of pathway‐based subtypes on single cell resolution

**FIGURE 5:**
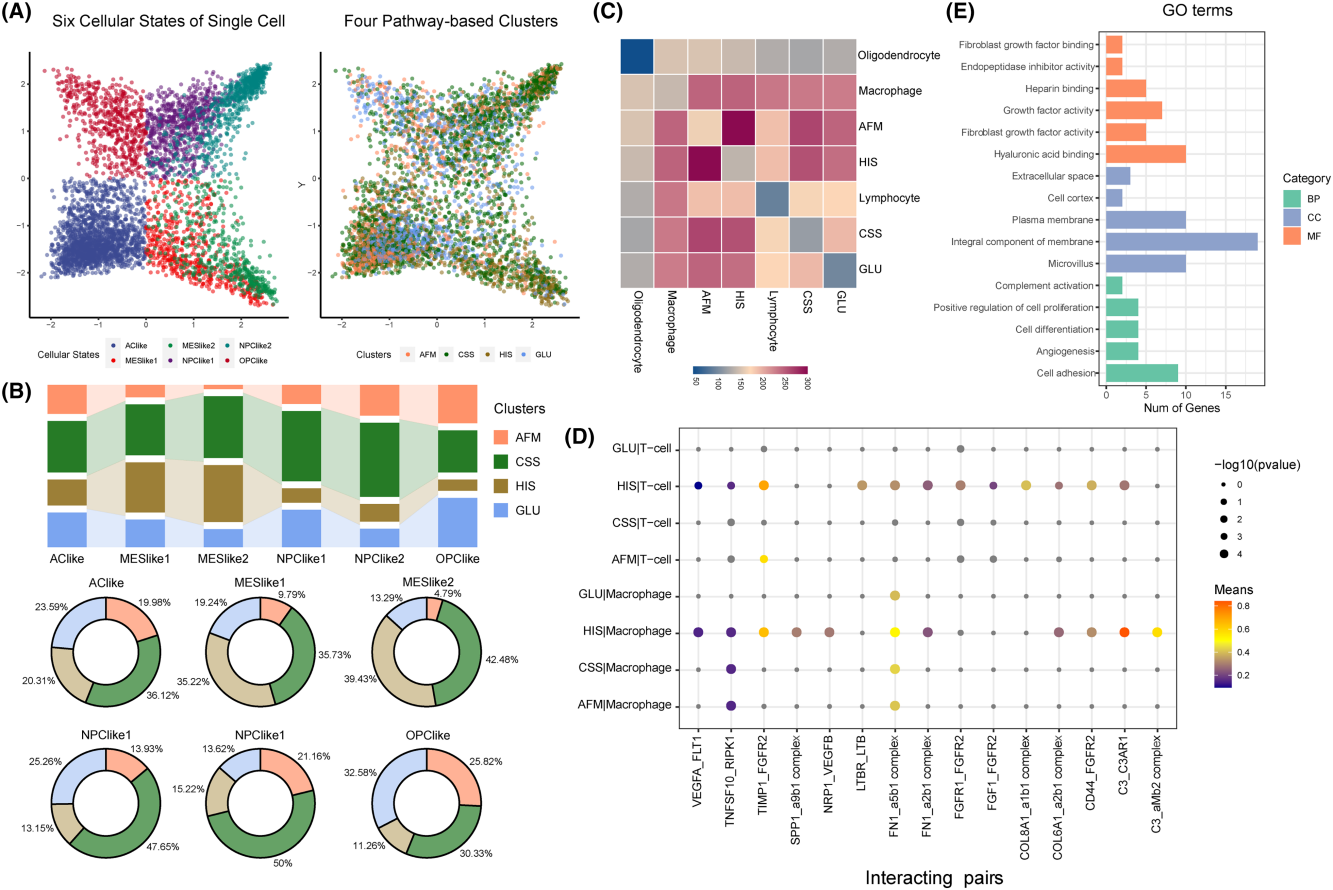
Validation of four functional clusters in single cell level. (A) Left: TSNE dimensionality reduction plot of tumour cell from cellular states. Right: TSNE plot of tumour cell coloured based on four pathway‐based clusters. (B) The fraction of four pathway‐based clusters across six cellular states. (C) Heatmap exhibited the count of significant L‐R pairs between cell types. (D) The dotplot indicated the specific interacted L‐R pairs between HIS tumour cells and immune cells. (E) Barplot depicting from a GO enrichment analysis of the genes involved in 15 L‐R pairs.

To evaluate the strength of pathway‐based classification, we assessed the intersection of the four clusters with the existing and widely used multiple cellular states in single cell resolution. The four pathway‐based clusters were closely correlated with cellular states (Figure 5A). We found increased fraction of HIS subtype GBM cells in MESlike1 and MESlike2 subtypes, compared with other four subtype, suggesting the mesenchymal identity and hypoxia/ immunosuppression activities were inseparable features in GBM. Meanwhile, the AFM subtype GBM cells were mostly excluded from the MESlike1 and MESlike2 subtypes (Figure 5B).

The glioma heterogeneity was driven by tumour genetic and microenvironment. To comprehensively depict the interactions between tumour microenvironment and four pathway‐based clusters, we analysed the communication between tumour cell and other cells. The significant ligand‐receptor (L‐R) interactions were identified by the CellPhoneDB software. Hundreds of specific molecular pairs mediating cell–cell interactions pairs were found between different cell types, and communication between tumour cell and immune cell was dominated (Figure 5C). We found 15 L‐R pairs specific interacted between HIS subtype cells and immune cells, including C3_C3AR1, TIMP1_FGFR2, C3_aMb2 complex, CD44_FGFR2, SPP1_a9b1 complex, NRP1_VEGFB, COL6A1_a2b1 complex, FN1_a2b1 complex, VEGFA_FLT1, FN1_a5b1 complex, TNFSF10_RIPK1, COL8A1_a1b1 complex, FGF1_FGFR2, FGFR1_FGFR2 and LTBR_LTB (Figure 5D). Meanwhile, the gene ontology analysis indicated that the genes in 15 L_R pairs mainly involved in cell proliferation and angiogenesis progress. Collectively, these results indicate that the interactions between HIS type tumour cells and immune cells can alter neoplastic glioma cells towards a more proliferative phenotype that associates with poor prognosis (Figure 5E).

### 3.6 Identification of a HIS‐related prognostic signature for gliomas

**FIGURE 6:**
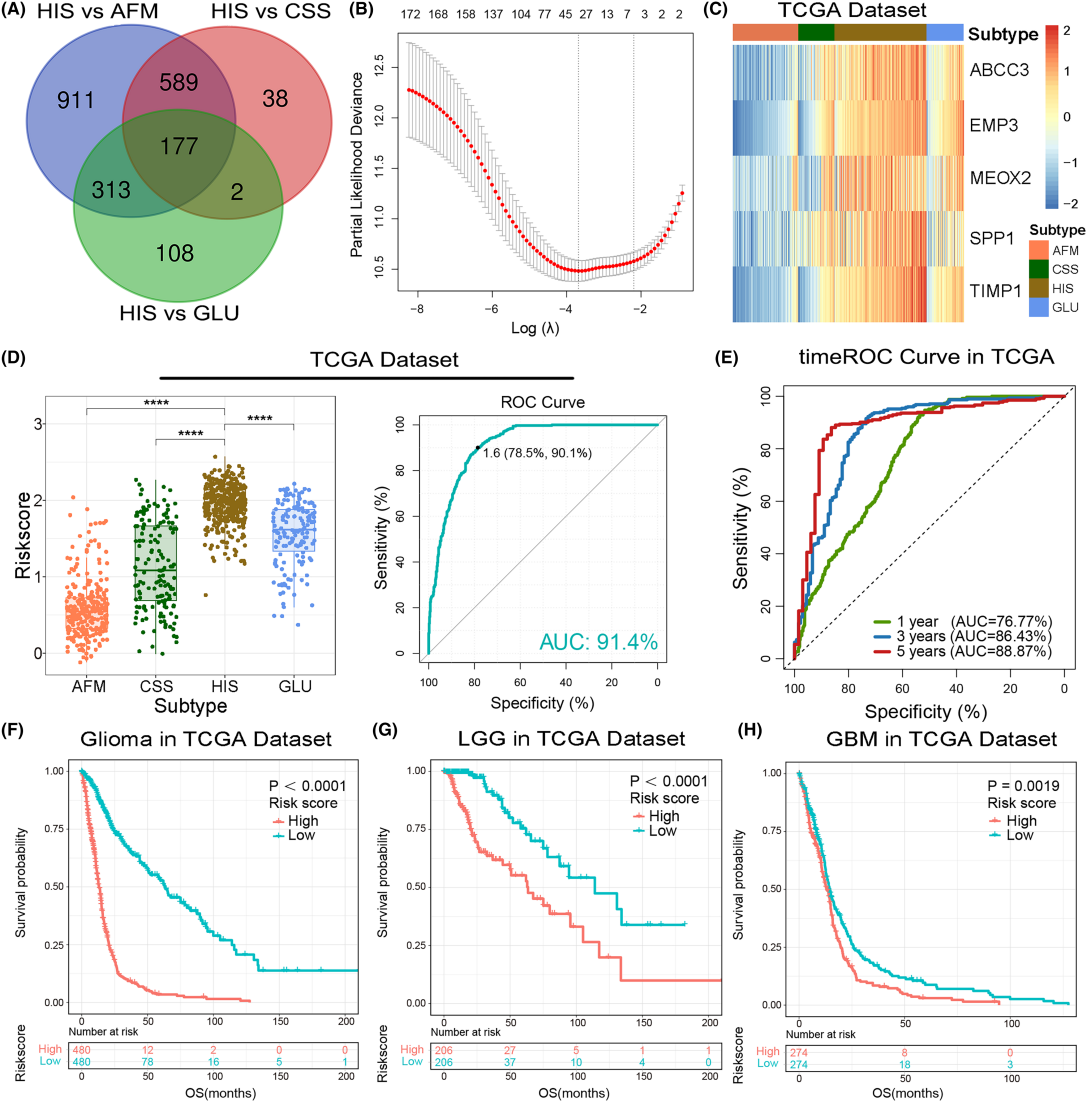
Identification of a risk signature by LASSO regression model. (A) Venn diagram shows the genes with the highest expression level in HIS among four subtypes. (B) Cross‐validation for tuning parameter selection in the LASSO regression model. (C) Heat map of 5 signature genes in TCGA data set. (D) The distribution of risk score in four TME‐related subtypes in TCGA data set. ROC curves predicted the risk signature as a biomarker of HIS subtype in TCGA data set. (E) In TCGA data set, the time ROC curve analyses suggested that the risk signature could accurately predict 1‐, 3‐, and 5‐year OS of glioma patients. (F–H) Survival analyses of the risk signature in the whole grade (F), LGG (G), and GBM (H) samples from TCGA data set.

According to the results mentioned above, glioma samples in HIS subtype had the characteristics of immunosuppression, oxygen deprivation and active energy metabolism, resulting in a poor clinical outcome. Therefore, to provide more reasonable and effective treatment strategies, it is necessary to identify patients in HIS subtype accurately. Here, we constructed a HIS‐related signature based on the differential genes among four clusters in TCGA data set for patient screening and prognosis prediction. The differentially expressed genes were identified by ‘limma’ package in R‐3.6.2, wherein 177 genes were significantly overexpressed in HIS subtype (Fold change >1, *p* < 0.05, Figure 6A). Then, LASSO Cox regression model was performed for to select genes with best prognostic value (Figure 6B, Supplementary Figure S6A). Subsequently, we constructed a five‐gene signature and the risk score of each sample was calculated using the formula mentioned in ‘Materials and Methods’ section (Figure 6C, Supplementary Figure S6B,C). After exploring the distribution of risk score among four clusters TCGA and CGGA data sets, we found that HIS subtype had the highest risk score (Figure 6D, Supplementary Figure S6D,F). Afterwards, ROC curve analysis was performed to assessed the predictive accuracy by computing area under the curve (AUC) of risk score. The AUC of risk score (91.4% in TCGA data set, 90.2% in CGGA data set1 and 91.9 in CGGA data set2) suggested that the risk signature could accurately predict glioma samples from HIS subtype, which has important reference value for individualized treatment of gliomas.

To evaluated the prognostic value of the risk signature, we performed timeROC analysis by ‘timereg’ package in TCGA and CGGA data sets. The results suggested the superior performance of this signature for prediction of 1‐, 3‐ and 5‐years survival rate (Figure 6E, Supplementary Figure S6E,G). Besides, samples from TCGA and CGGA data sets were divided into low‐ or high‐risk group according to the median value of risk scores. Kaplan–Meier (K–M) survival analysis revealed that patients in low‐risk group had a better prognosis than those in high‐risk group (Figure 6F, Supplementary Figure S7A,B). Considering the significant difference of pathological characteristics between lower‐grade glioma (LGG, WHO Grade 2 and 3) and GBM, we also performed K‐M survival analysis in LGG and GBM, respectively. Consistently, the survival curve showed that both LGG and GBM patients in low‐risk group tended to have a better clinical outcome. (Figure 6G,H, Supplementary Figure S7C–F). Furthermore, univariate and multivariate Cox regression analyses was performed to evaluated the prognostic independence of this risk signature. As expected, the signature was a prognostic factor independent of other clinicopathological features in gliomas in TCGA and CGGA data sets (Table 1, Supplementary Tables S3 and S4). These results demonstrated that the HIS‐related signature had great value of clinical practice for patient screening and prognosis prediction.

**TABLE 1:** Univariate and multivariate analysis of OS in TCGA data set.

Variables	Univariate analysis		Multivariate analysis	
	HR (95% CI)	*p* value	HR (95% CI)	*p* value
Risk score	4.622 (3.879–5.508)	<0.001	1.833 (1.112–3.023)	0.017
Age at Diagnosis	1.057 (1.050–1.065)	<0.001	1.607 (1.194–2.163)	0.002
Gender	1.115 (0.930–1.336)	0.239	–	–
WHO Grade	4.005 (3.317–4.836)	<0.001	1.803 (1.266–2.567)	0.001
TCGA Subtype	1.531 (1.394–1.681)	<0.001	0.950 (0.830–1.088)	0.460
IDH mutation status	0.124 (0.094–0.162)	<0.001	0.865 (0.462–1.622)	0.652
MGMT methylation	0.368 (0.295–0.458)	<0.001	0.823 (0.627–1.078)	0.158
1p19q co‐deletion	0.147 (0.091–0.239)	<0.001	0.535 (0.295–0.968)	0.039

### 3.7 Functional annotation and clinical application of the risk signature

**FIGURE 7:**
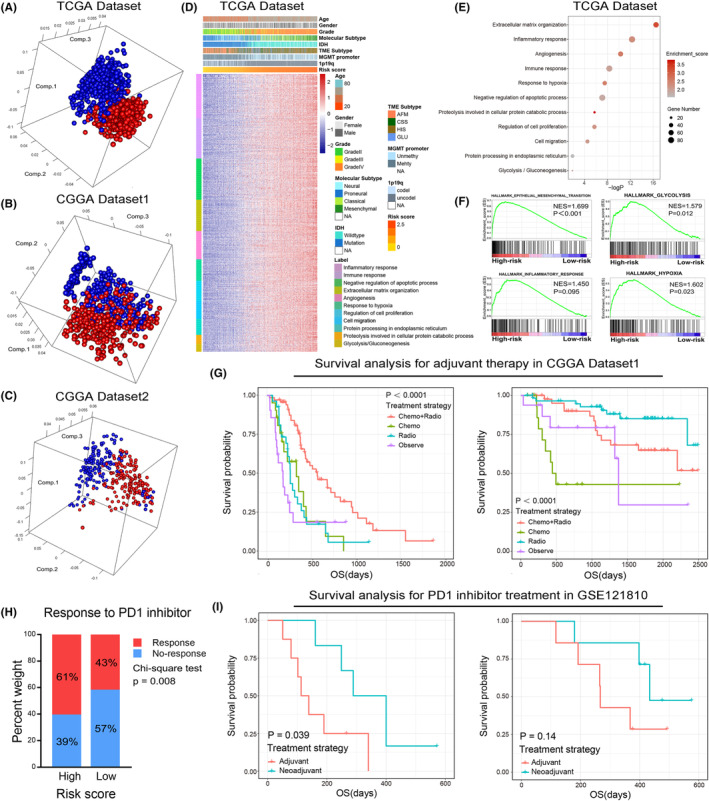
The biological functions and clinical applications of the risk signature. (A–C) PCA of whole gene expression data between high‐risk and low‐risk groups in TCGA and CGGA data sets. (D, E) GO functional analyses were performed to explore the functional annotation of the risk signature in TCGA data set. (F) GSEA was performed to explore the biological functions that were positively correlated to risk score in TCGA data set. (G) Kaplan–Meier survival curve analysis for glioma patients with different postoperative adjuvant therapy in high‐risk and low‐risk group. (H) Rate of clinical response to anti‐PD1 immunotherapy in high‐ or low‐risk groups in the cohort deposited in SRA PRJNA482620 (Chi‐square test, *p* = 0.008). (I) Kaplan–Meier survival curve analysis for glioma patients with different PD1 inhibitor treatment strategies in high‐risk and low‐risk group of GSE121810 data set.

In both TCGA and CGGA data sets, PCA showed that patients in the high‐risk and low‐risk group tended to have differential gene expression profiles (Figures 7A–C). Thus, we hypothesize that samples in the low‐risk and high‐risk group have different biological features. To verify this, Pearson's correlation analysis was performed to select genes that positively correlated with the risk score (*R* > 0.5) in TCGA and CGGA data sets. Then, these genes were uploaded to DAVID website for functional annotation. The GO analysis revealed that the risk score was positively correlated with immune and inflammatory response, response to hypoxia, glycolysis and protein metabolic processes and negatively correlated with apoptotic process (Figure 7D,E, Supplementary Figure S8A–C). Besides, we also found that the extracellular matrix organization, angiogenesis, cell proliferation and migration were significantly enriched in the high‐risk group, suggesting the risk signature was tightly associated with the malignant phenotype of gliomas (Figure 7D,E, Supplementary Figure S8A–C). To improve accuracy, GSEA was performed in TCGA data set as a validation and similar results were uncovered (Figure 7F). These results indicated that the risk signature derived from the new classification based on the immunologic, hypoxia and metabolic status could represent the malignant progress of gliomas.

To evaluate the clinical application of the risk signature, we focussed on the effect of the risk score on the efficacy of postoperative adjuvant therapy. As mentioned before, we divided patients into high‐risk and low‐risk groups according to the risk score. The K‐M survival analysis revealed that compared with other treatment strategies, postoperative radiotherapy combined with chemotherapy can significantly improve the prognosis of patients in high‐risk group (Figure 7G, left). However, for patients in low‐risk group, postoperative adjuvant therapy could not significantly improve the clinical outcome (Figure 7G, right). These results implied that patients in high‐risk group can benefit significantly from postoperative adjuvant therapy, and more aggressive treatment strategies should be taken. Moreover, we also found that patients in high‐risk group and HIS subtypes have a higher probability of responding to anti‐PD1 immunotherapy (Figure 7H and Supplementary Figure S8D, *p* = 0.008 and *p* = 0.0443, respectively). To find an optimal immunotherapy strategy for patients with gliomas. GSE121810 data set was enrolled in this study.^22^ The K‐M survival analysis suggested that neoadjuvant administration of PD‐1 blockade could significantly prolong the OS of patients in high‐risk group but not the low‐risk group (Figure 7I). Thus, the risk signature might serve as an important reference in individualized treatments for glioma patients.

## 4 DISCUSSION

As the most prevalent and lethal primary brain tumours in adults, gliomas account for 80% of all malignant brain tumours.^41^ Due to the intricate TME and heterogeneity of glioma cells, the therapeutic efficacy of conventional treatment was limited.^42^ The TME iskey regulator of tumour genesis, proliferation, invasion and metastasis.^5^ Considering the widely varying therapeutic efficacy and clinical outcomes of gliomas, it is essential to propose a robust classifier to stratify patients with different TME features and prognosis, which is of great importance to maximize the benefits of individualized treatment strategy.

In the present study, we comprehensively analysed the microenvironment characteristics of glioma samples based on the transcriptome data from TCGA and CGGA data sets and tried to construct a tool to figure out this important clinical issue.

The TME not only comprises cancer cells but also composed of stromal cells (including immune cells, endothelial cells and fibroblasts), the extracellular matrix (ECM) and a variety of cytokines and metabolites.^43^ In addition, a wide range of physicochemical factors, such as oxygen content, PH value and ion concentration are also integral components of TME.^7^, ^10^ In the past decade, it has become evident that the TME actively participates in genesis and malignant progress of cancers.^5^ In the early beginning of tumorigenesis, immune cells were recruited and infiltrated into the TME and profoundly impact the progression, invasion and drug resistance of cancers.^44^, ^45^ For example, as a key component of the TME, the M2 type macrophage have pro‐tumorigenic and immunosuppressive properties in most cases.^46^ In addition, the CD8+ T cells, which were considered to have significant antitumor effect tend to functional exhaustion in TME of gliomas.^47^ In this study, we also found that the M2 macrophages and memory CD4+ T cells tend to enriched in glioma samples of HIS subtype, which eventually leads to poor prognosis. It is worth mentioning that the immunotherapy, especially the targeting PD‐1/PD‐L1, has achieved marked success in multiple human cancers,^48^, ^49^ and it also has a potential clinical value in the treatment of gliomas. Considering the high expression level of multiple immune checkpoints in the samples of HIS subtype, these patients may benefit from immunotherapy, which has also been initially proved in this study.

However, immune response is not the only factor that determines the fate of gliomas. As tumours grow, the dysfunctional tumour‐neovascular vessels fail to sufficiently perfuse the tumour, which creates a hypoxic and nutrient deficient tumour microenvironment.^15^ Tumour hypoxia confers drug resistance and lead to a poor prognosis of patients with gliomas. With the accumulation of metabolic byproducts and immunosuppressive factors, hypoxic TME triggers angiogenesis, cell metabolic reprogramming and shape the infiltration and phenotype of immune cells.^15^ Importantly, glioma stem‐like cells (GSCs), which have a high capacity for self‐renewal and therapeutic resistance, tend to reside in a hypoxic niche to maintain their biological functions.^7^ Thus, considering the essential role of hypoxia in TME, hypoxia‐driven signals were the key to fully understand the biology of TME in gliomas. By performed bioinformatic analysis and IHC analysis, we found that there were significant differences in hypoxia status among the four subtypes of gliomas. It means that the pathway‐based subtypes are of high value in evaluating the hypoxic state of glioma samples.

The biological behaviour of cancer cells and immune cells are also tightly linked to metabolic profiles. In the hypoxic and nutrient scarcity microenvironment, the metabolic competition becomes more intense.^39^ However, adaptive changes in cancer cells that allow them to survive in this harsh condition. It is widely known that cancer cell prefers glycolysis to sustain their high proliferation needs, even if it was less efficiency than oxidative phosphorylation (OXPHOS).^50^, ^51^ In colorectal cancer, the mutation of KRAS or BRAF genes could increase the expression level of glucose transporter‐1 (GLUT1) and enhance glycolysis.^52^ In line with this, the loss of PTEN could promote glucose uptake and consumption in melanomas.^53^ Our research also showed that HIS subtype with the high malignancy has the most active glucose and nucleotides metabolism. However, for immune cells, hypoxia and scarce nutrient availability may induce the activation of AMPK signalling pathways, which enhances OXPHOS and induces immunosuppressive phenotype of these cells.^54^ For example, M2 macrophages, which plays a key role in the malignant progression of glioma, have a higher rate of OXPHOS than M1 macrophages.^55^, ^56^ Thus, the TME, including factors such as hypoxia, immune response and cell metabolism states, has a decisive role in the tumorigenesis and progression of cancers.

In the present study, to comprehensively estimate the functional roles of TME in gliomas, we divided glioma samples into four subtypes based on the hypoxia responses, immune responses and metabolism‐related pathways. We found that the TME phenotypes were tightly correlated with the genomic characteristics, clinicopathological features and overall survival time of patients with gliomas. It is worth mentioning that IDHmt samples can be mainly divided into AFM and CSS subtypes, while most IDHwt samples belong to HIS and GLU subtypes (Figure 1F), suggesting that glioma samples with same IDH mutation status may have different TME features. Besides, we also identified a subgroup of patients characterized by intensive hypoxia, immunosuppression and poor clinical outcome (HIS subtype). The scRNA‐seq analysis indicated that HIS type GBM cells mainly enriched in the MESlike1 and MESlike2 subtype. On this foundation, we preliminarily explored the molecular mechanism of the interaction between HIS type cells and immune cells, which lead to malignant progression of gliomas and identified 15 ligand‐receptor pairs. This provides a new insight into the intercellular interactions in the microenvironment of gliomas. In gliomas, abundant immunocyte infiltration is often tightly associated with a higher degree of tumour malignancy and an unfavourable prognosis.^13^, ^57^ The immune microenvironment characteristics of the HIS subtype further confirmed this point. We hypothesized that there are the following reasons for this phenomenon: In gliomas, tumour‐infiltrating T cells usually exhibit an exhaustive phenotype, and the increased presence of regulatory T cells also promotes immune suppression.^58^ Additionally, the substantial infiltration of tumour‐associated macrophages (TAMs) significantly promotes the malignant progression of gliomas.^12^, ^56^ Furthermore, immune cell infiltration is typically accompanied by an inflammatory response, and chronic inflammation may facilitate the growth and invasion of the tumour.^59^

To provide more accurate diagnosis and effective treatment strategies, we constructed a HIS‐related signature. The results suggested that the risk signature was an independent prognostic factor and patients in high‐risk group need mor aggressive treatment strategies. We also found that patients in high‐risk group were more sensitive to neoadjuvant PD‐1 targeted therapy, which provides an important reference for immunotherapy of gliomas. The correlation between risk score and response to anti‐PD1 immunotherapy provided important guiding significance for immunotherapy in glioma patients and was worthy of further validation and discussion in future researches. Considering that the risk signature was performed to identify patients in HIS subtype. It means that samples in the high‐risk group are more likely to have molecular pathological features consistent with HIS subtypes, such as high expression of several immune checkpoints and significant immunosuppression. This may be the main reason why patients in high‐risk group would benefit more from anti‐PD‐1 therapy. However, this hypothesis needs more rigorous validation in subsequent researches.

In conclusion, our results showed that gliomas could be further classified into four TME‐related subtypes, which have different TME characteristics and significant prognostic differences, providing key references for individualized treatment of gliomas. Although, these findings provided a reasonable stratification of patients with gliomas and represent a new insight into TME characteristics, the results of our study should be further validated by prospective cohorts and functional experiments. Considering the different biological behaviours of LGG and GBM, it is worthwhile to further explore the pathway‐based subtypes in LGG and GBM respectively in the following study.

## AUTHOR CONTRIBUTIONS

**Ruoyu Huang:** Conceptualization (equal); formal analysis (equal); methodology (equal); software (equal); writing – original draft (equal). **Bo Han:** Formal analysis (equal); methodology (equal); resources (equal); writing – original draft (equal); writing – review and editing (equal). **Ying Zhang:** Formal analysis (equal); writing – original draft (equal). **Jingchen Yang:** Methodology (equal); resources (equal). **Kuanyu Wang:** Formal analysis (equal); project administration (equal). **Zhiliang Wang:** Conceptualization (equal); methodology (equal); supervision (equal). **Xing Liu:** Data curation (equal); methodology (equal); supervision (equal).

## FUNDING INFORMATION

This work was supported by grants from the National Natural Science Foundation of China (81902533, 82102764 and 82003192).

## CONFLICT OF INTEREST STATEMENT

The authors declare that there are no conflicts of interest.

## Supporting information


Supplementary Figure S1.

## Data Availability

All data supporting this study were openly available.
