# Supplementary material for: Pathway‐based stratification of gliomas uncovers four subtypes with different TME characteristics and prognosis
Source: J Cell Mol Med. 2024 Apr 13;28(8):e18208. doi: 10.1111/jcmm.18208 (PMC11015396; doi:10.1111/jcmm.18208)
Supplement: Supplementary file 1 — Supplementary Figure S1. [file JCMM-28-e18208-s001.docx]

**Supplementary Materials**

**Supplementary Figure 1**

**
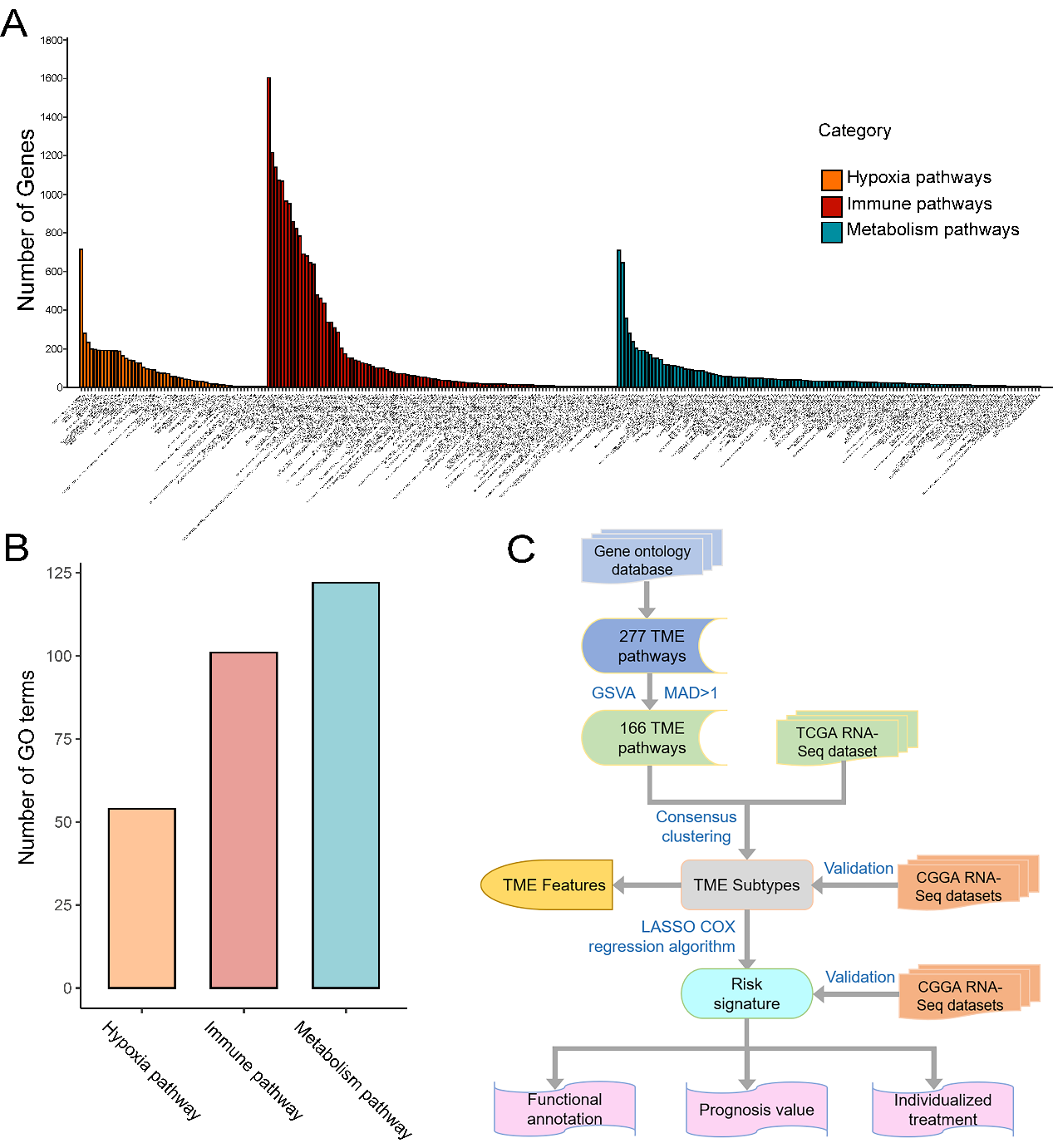
Supplementary Figure 1.** Overview of 277 TME-related signaling pathways (A, B) and workflow of this study (C).

**Supplementary Figure 2**

**
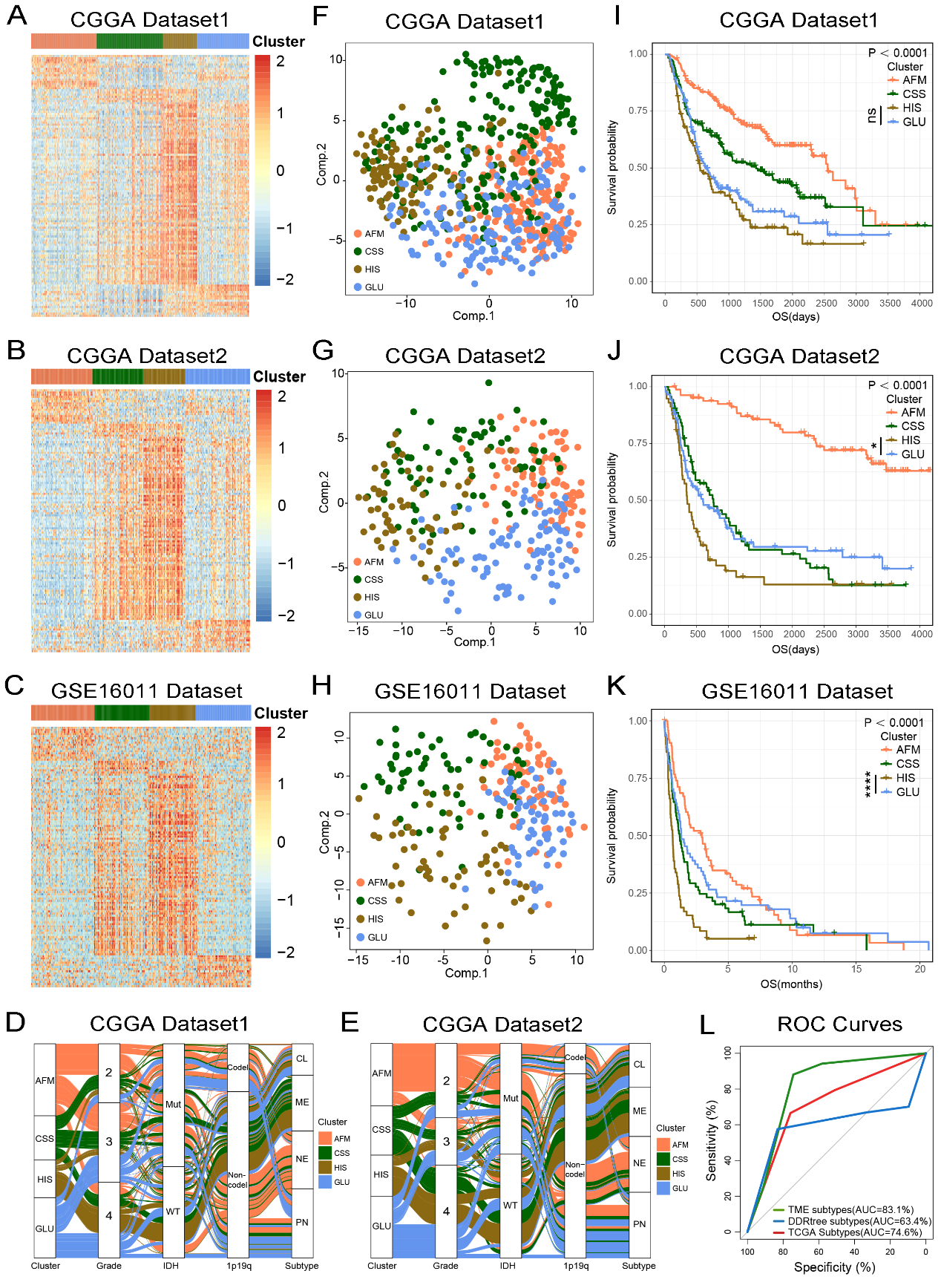
**

**Supplementary Figure 2.** Validation of reproducibility of four TME-related subtypes in gliomas in CGGA and GSE16011 datasets.

(A-C) Heatmap of four subtypes defined by TME-related signaling pathways in CGGA datasets. (D, E) Sankey diagram exhibiting the association between cluster and clinicopathological characteristics of glioma samples in CGGA datasets. (F-H) PCA analysis of four subtypes in CGGA and GSE16011 datasets. (I-K) Kaplan–Meier analysis of four clusters based on overall survival (OS) in CGGA and GSE datasets. (L) ROC curves analyses were performed to estimate ability of three subtypes in predicting patients’ survival in TCGA cohort.

**Supplementary Figure 3**

**
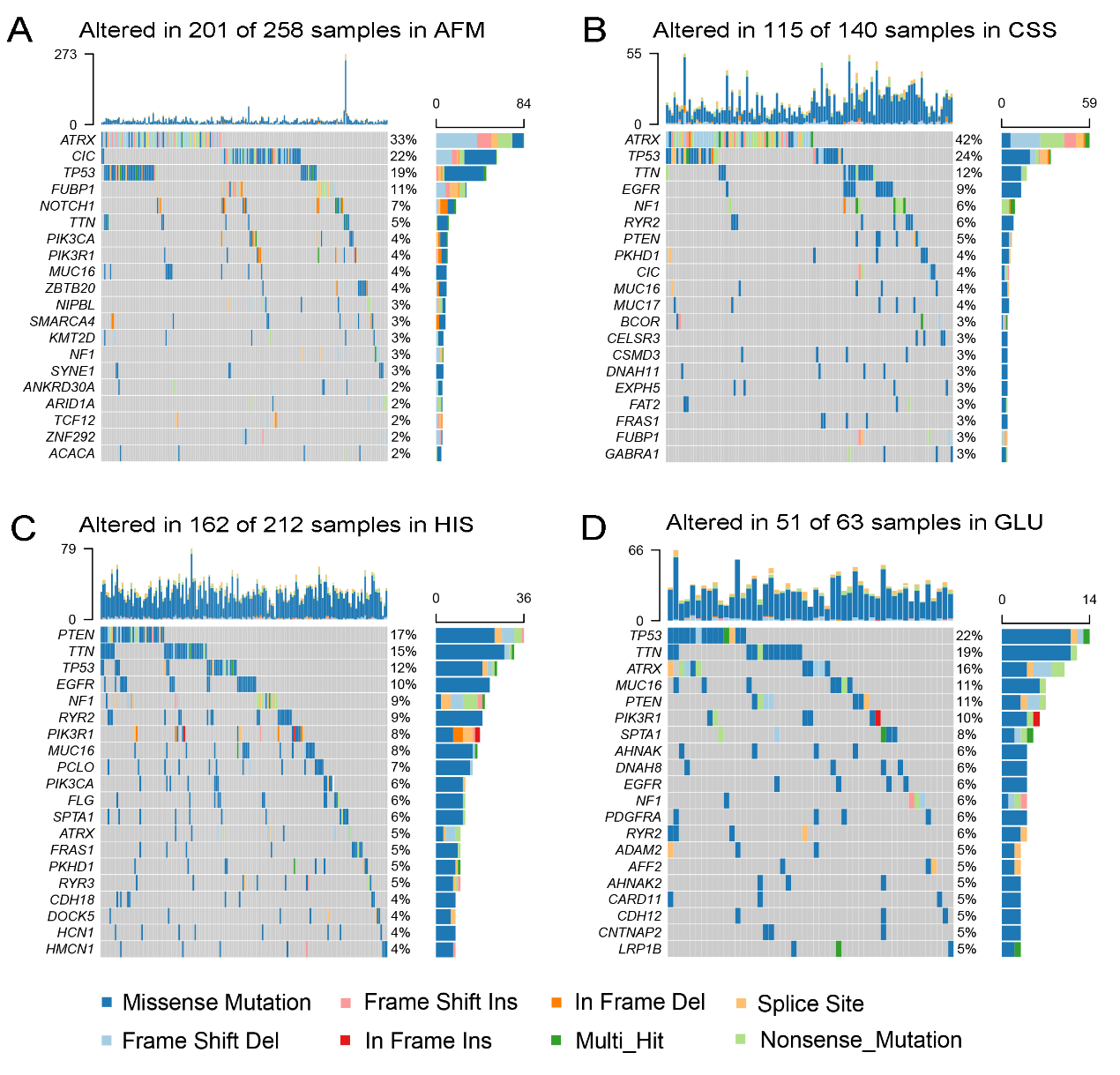
**

**Supplementary Figure 3.** The landscape of somatic mutation in four subtypes.

**Supplementary Figure 4**

**
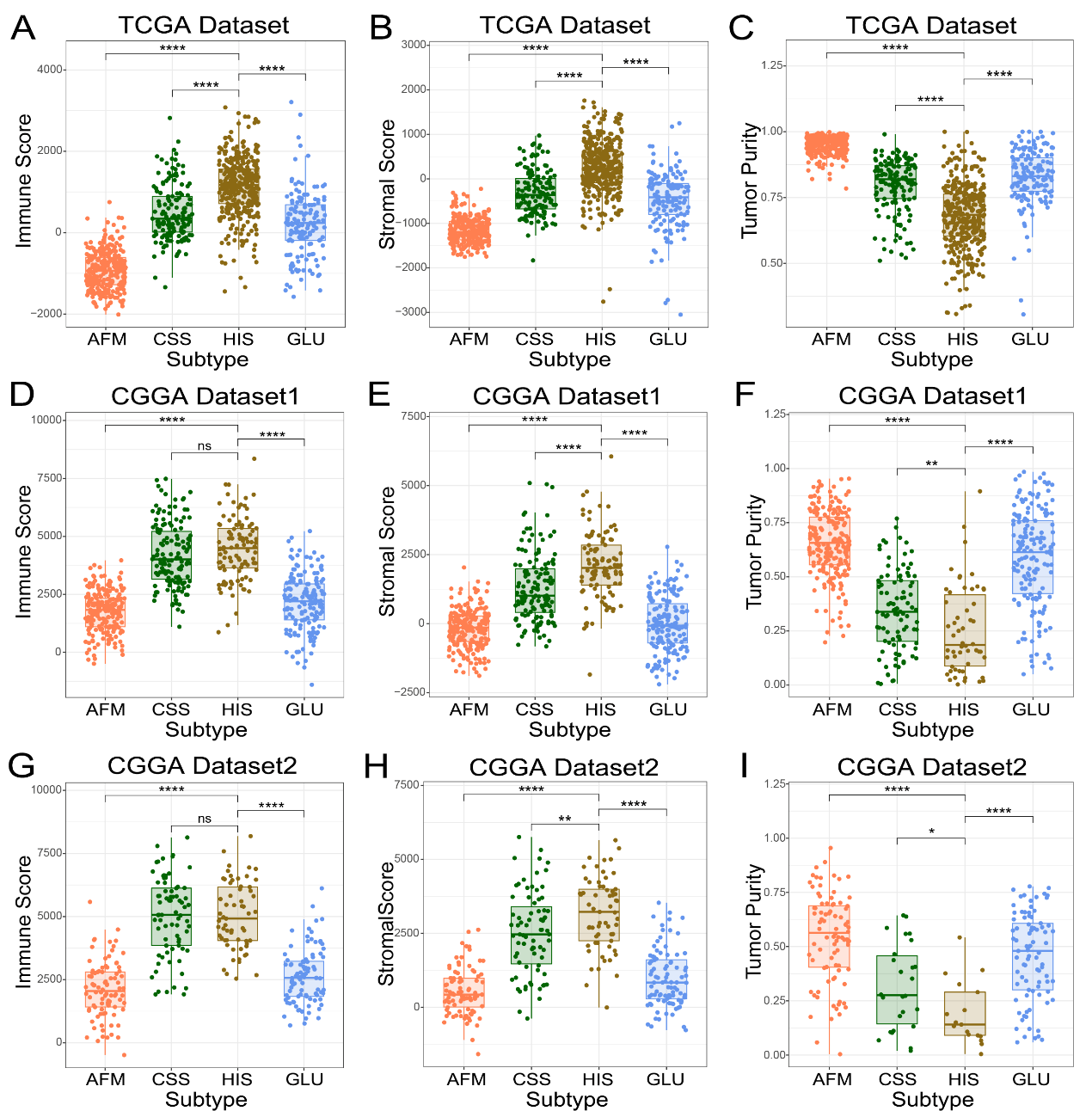
**

**Supplementary Figure 4.** The distribution of immune score, stromal score and tumor purity in TCGA dataset (A-C), CGGA dataset1 (D-F) and CGGA dataset2 (G-I), respectively.

**Supplementary Figure 5**


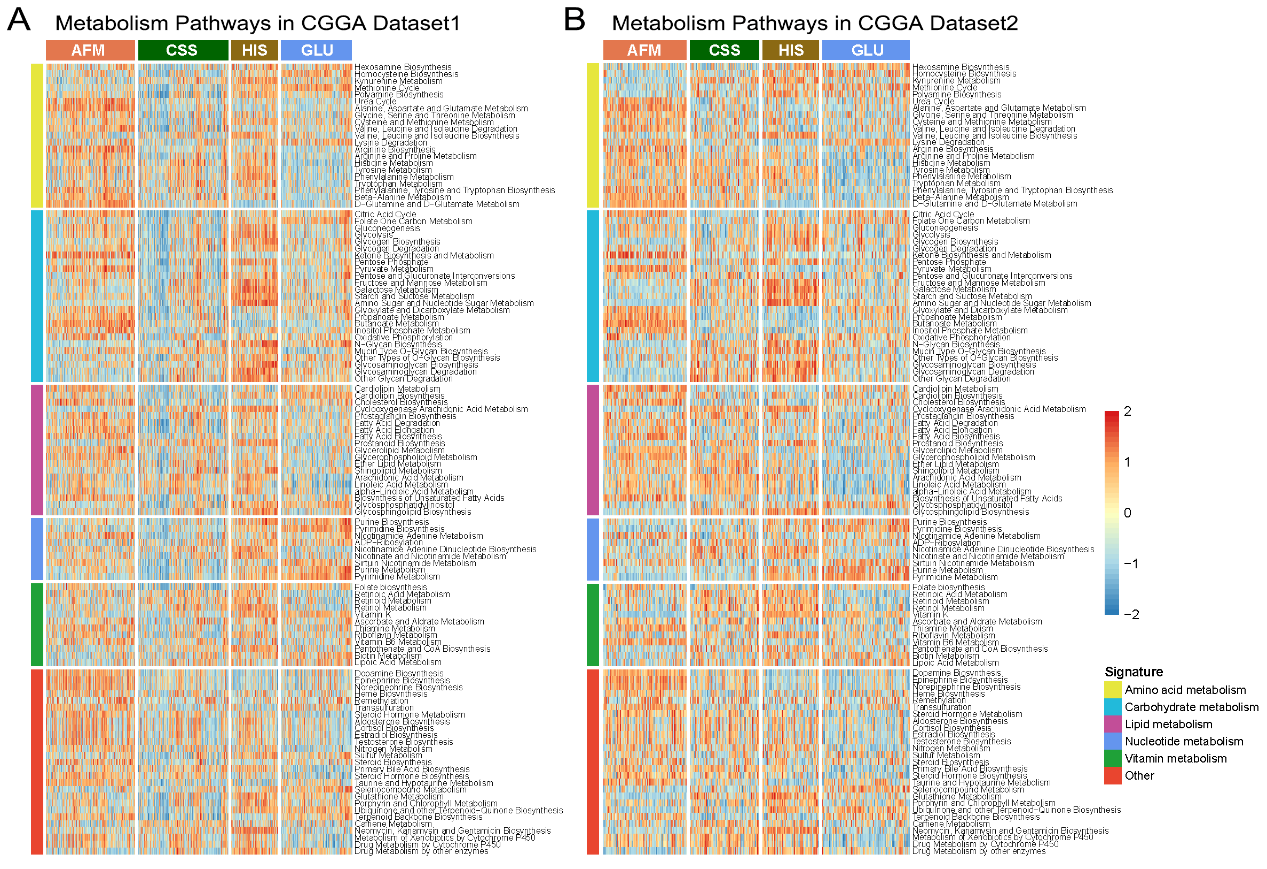


**Supplementary Figure 5.** The differences in the activation of cellular metabolic pathways among four subtypes in CGGA dataset1 (A) and CGGA dataset2 (B).

**Supplementary Figure 6**

**
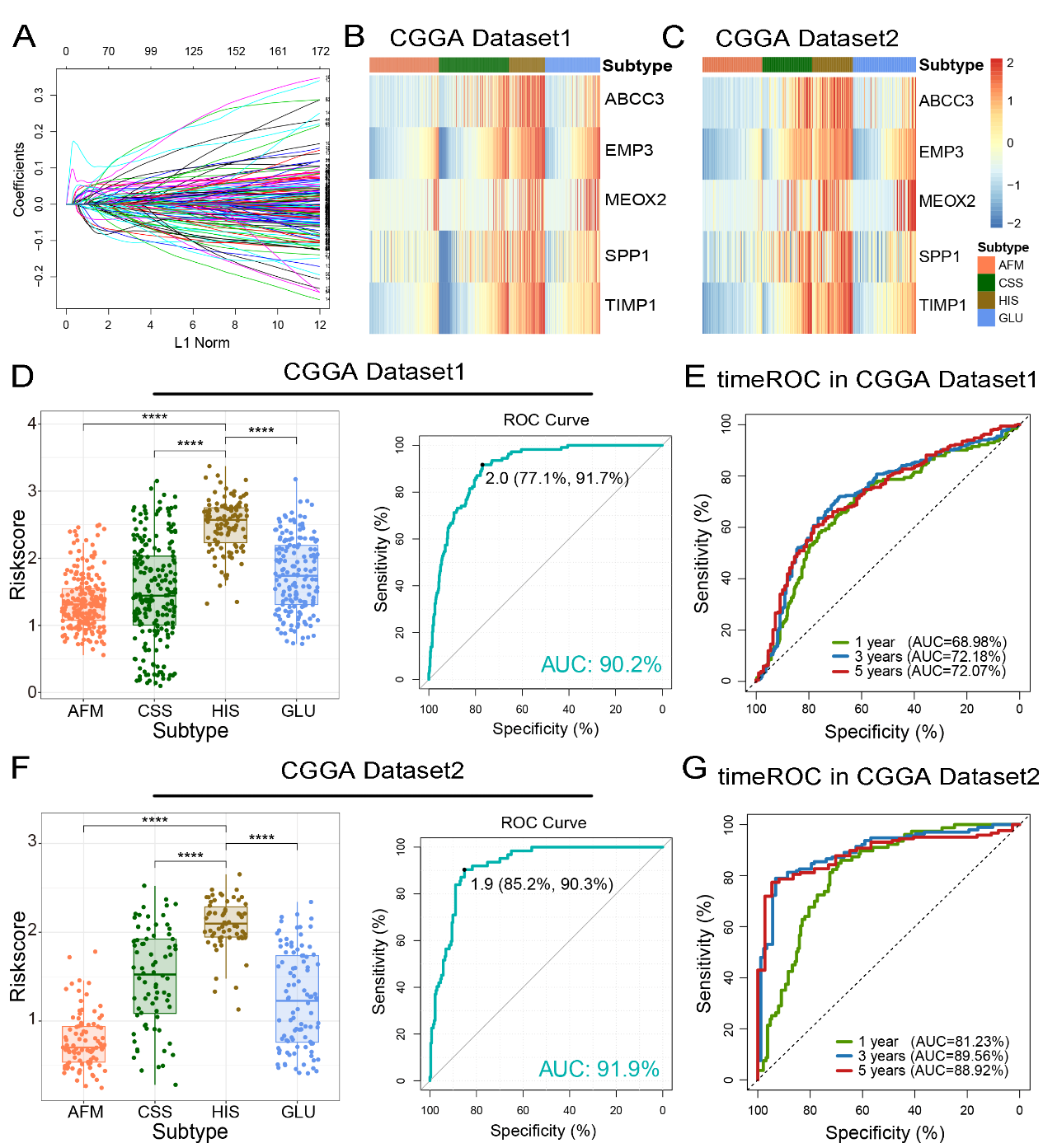
**

**Supplementary Figure 6.** Validation of reproducibility of the risk signature in CGGA datasets.

(A) The coefficient profiles of LASSO regression model. (B, C) Heat map of 5 signature genes in CGGA datasets. (D, F) The distribution of risk score in four TME-related subtypes in CGGA datasets. ROC curves predicted the risk signature as a biomarker of Cluster3 in CGGA datasets. (E, G) in CGGA datasets, the time ROC curve analyses suggested that the risk signature could accurately predict 1-, 3-, and 5-year OS of glioma patients.

**Supplementary Figure 7**

**
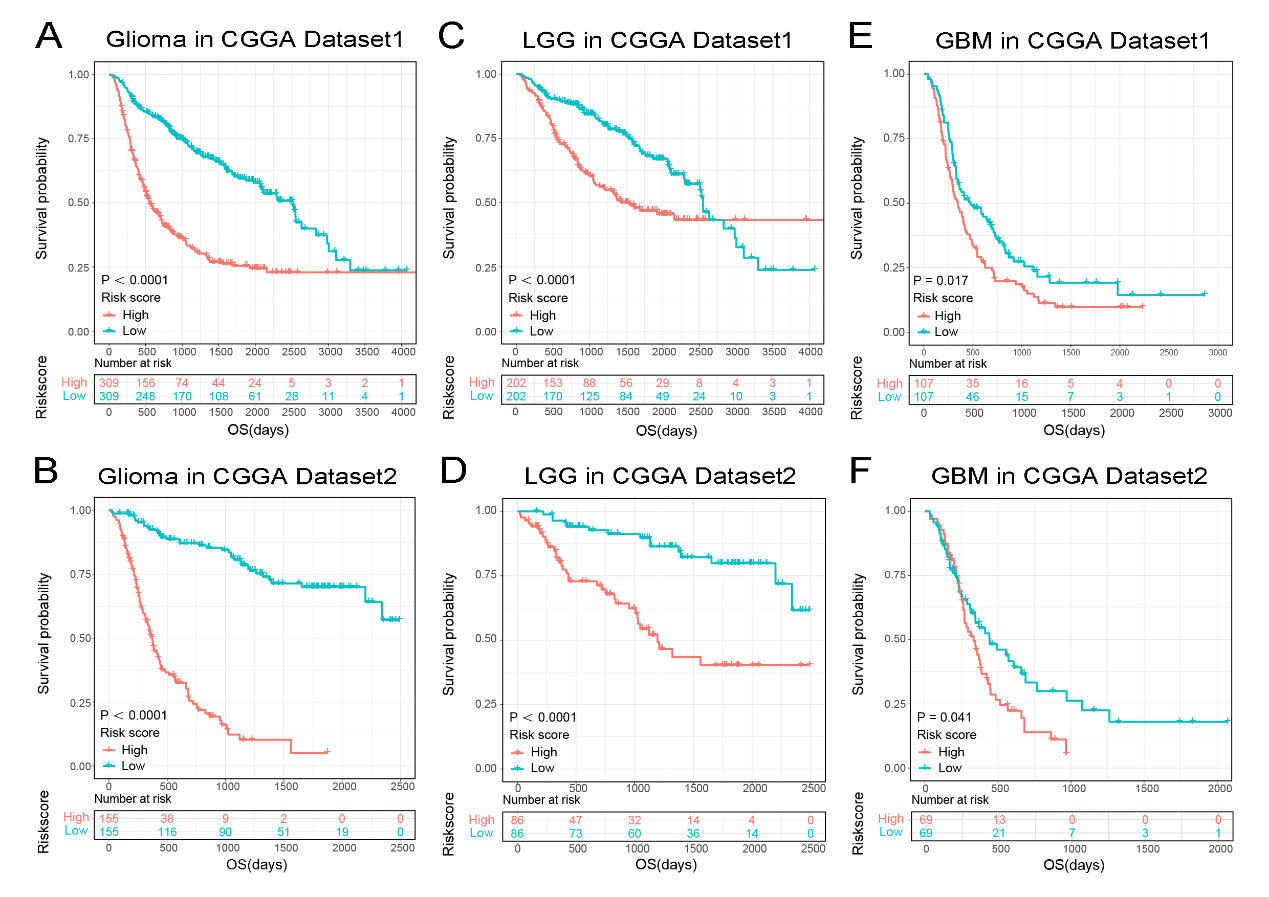
**

**Supplementary Figure 7.** Survival analyses of the risk signature in the whole grade (A, B), LGG (C, D), and GBM (E, F) samples from CGGA datasets.

**Supplementary Figure 8**

**
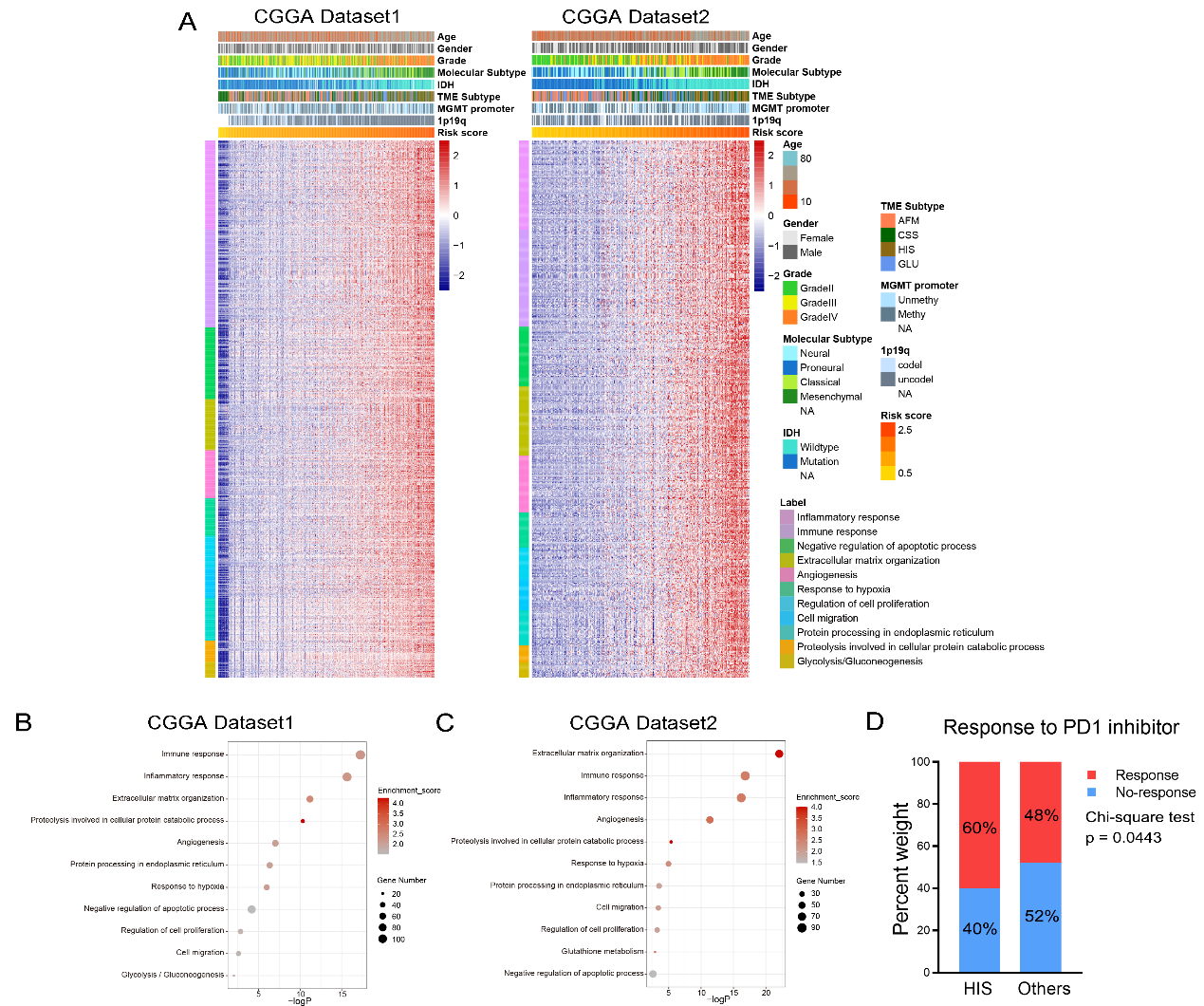
**

**Supplementary Figure 8.** The biological functions and clinical applications of the risk signature.

(A-C) GO functional analyses were performed to explore the functional annotation of the risk signature in CGGA datasets. (D) Rate of clinical response to anti-PD1 immunotherapy in HIS or ohter subtypes in the cohort deposited in SRA PRJNA482620 (Chi-square test, P = 0.0443).

**Supplementary Table 1.** 277 TME-related pathways.

| **TME Pathways** | **Category** | **Gene Counts** |
| --- | --- | --- |
| KRIEG_HYPOXIA_NOT_VIA_KDM3A | Hypoxia | 744 |
| MANALO_HYPOXIA_DN | Hypoxia | 292 |
| WINTER_HYPOXIA_METAGENE | Hypoxia | 242 |
| GROSS_HYPOXIA_VIA_ELK3_UP | Hypoxia | 208 |
| MANALO_HYPOXIA_UP | Hypoxia | 205 |
| JIANG_HYPOXIA_NORMAL | Hypoxia | 201 |
| KRIEG_KDM3A_TARGETS_NOT_HYPOXIA | Hypoxia | 200 |
| HALLMARK_HYPOXIA | Hypoxia | 200 |
| GSE22282_HYPOXIA_VS_NORMOXIA_MYELOID_DC_UP | Hypoxia | 200 |
| GSE22282_HYPOXIA_VS_NORMOXIA_MYELOID_DC_DN | Hypoxia | 199 |
| GSE26023_PHD3_KO_VS_WT_NEUTROPHIL_HYPOXIA_UP | Hypoxia | 198 |
| GSE26023_PHD3_KO_VS_WT_NEUTROPHIL_HYPOXIA_DN | Hypoxia | 196 |
| ELVIDGE_HYPOXIA_UP | Hypoxia | 172 |
| GROSS_HYPOXIA_VIA_ELK3_DN | Hypoxia | 156 |
| ELVIDGE_HYPOXIA_DN | Hypoxia | 147 |
| GROSS_HYPOXIA_VIA_ELK3_AND_HIF1A_UP | Hypoxia | 143 |
| ELVIDGE_HYPOXIA_BY_DMOG_UP | Hypoxia | 133 |
| QI_HYPOXIA | Hypoxia | 132 |
| GROSS_HYPOXIA_VIA_HIF1A_DN | Hypoxia | 109 |
| GROSS_HYPOXIA_VIA_ELK3_AND_HIF1A_DN | Hypoxia | 101 |
| MENSE_HYPOXIA_UP | Hypoxia | 97 |
| WINTER_HYPOXIA_UP | Hypoxia | 95 |
| HARRIS_HYPOXIA | Hypoxia | 81 |
| GO_REGULATION_OF_TRANSCRIPTION_FROM_RNA_POLYMERASE_II_PROMOTER_IN_RESPONSE_TO_HYPOXIA | Hypoxia | 77 |
| GROSS_HYPOXIA_VIA_HIF1A_UP | Hypoxia | 76 |
| REACTOME_CELLULAR_RESPONSE_TO_HYPOXIA | Hypoxia | 75 |
| ELVIDGE_HYPOXIA_BY_DMOG_DN | Hypoxia | 59 |
| JIANG_HYPOXIA_CANCER | Hypoxia | 58 |
| KRIEG_HYPOXIA_VIA_KDM3A | Hypoxia | 53 |
| WINTER_HYPOXIA_DN | Hypoxia | 51 |
| GROSS_HYPOXIA_VIA_ELK3_ONLY_DN | Hypoxia | 44 |
| LEONARD_HYPOXIA | Hypoxia | 43 |
| WEINMANN_ADAPTATION_TO_HYPOXIA_DN | Hypoxia | 39 |
| QI_HYPOXIA_TARGETS_OF_HIF1A_AND_FOXA2 | Hypoxia | 35 |
| GROSS_HYPOXIA_VIA_ELK3_ONLY_UP | Hypoxia | 34 |
| FARDIN_HYPOXIA_11 | Hypoxia | 32 |
| WEINMANN_ADAPTATION_TO_HYPOXIA_UP | Hypoxia | 27 |
| BIOCARTA_P53HYPOXIA_PATHWAY | Hypoxia | 21 |
| KIM_HYPOXIA | Hypoxia | 19 |
| JIANG_HYPOXIA_VIA_VHL | Hypoxia | 19 |
| GO_REGULATION_OF_CELLULAR_RESPONSE_TO_HYPOXIA | Hypoxia | 15 |
| WACKER_HYPOXIA_TARGETS_OF_VHL | Hypoxia | 13 |
| MIZUKAMI_HYPOXIA_UP | Hypoxia | 12 |
| REACTOME_REGULATION_OF_GENE_EXPRESSION_BY_HYPOXIA_INDUCIBLE_FACTOR | Hypoxia | 11 |
| KONDO_HYPOXIA | Hypoxia | 8 |
| GROSS_HYPOXIA_VIA_HIF1A_ONLY | Hypoxia | 8 |
| FARDIN_HYPOXIA_9 | Hypoxia | 7 |
| GO_NEGATIVE_REGULATION_OF_CELLULAR_RESPONSE_TO_HYPOXIA | Hypoxia | 7 |
| GO_INTRINSIC_APOPTOTIC_SIGNALING_PATHWAY_IN_RESPONSE_TO_HYPOXIA | Hypoxia | 7 |
| MAINA_HYPOXIA_VHL_TARGETS_UP | Hypoxia | 6 |
| MIZUKAMI_HYPOXIA_DN | Hypoxia | 6 |
| GO_POSITIVE_REGULATION_OF_TRANSCRIPTION_FROM_RNA_POLYMERASE_II_PROMOTER_IN_RESPONSE_TO_HYPOXIA | Hypoxia | 6 |
| GO_HYPOXIA_INDUCIBLE_FACTOR_1ALPHA_SIGNALING_PATHWAY | Hypoxia | 6 |
| GO_NEGATIVE_REGULATION_OF_HYPOXIA_INDUCED_INTRINSIC_APOPTOTIC_SIGNALING_PATHWAY | Hypoxia | 5 |
| GO_REGULATION_OF_IMMUNE_SYSTEM_PROCESS | Immune response | 1670 |
| GO_IMMUNE_EFFECTOR_PROCESS | Immune response | 1267 |
| GO_POSITIVE_REGULATION_OF_IMMUNE_SYSTEM_PROCESS | Immune response | 1187 |
| GO_REGULATION_OF_IMMUNE_RESPONSE | Immune response | 1116 |
| REACTOME_INNATE_IMMUNE_SYSTEM | Immune response | 1113 |
| GO_INNATE_IMMUNE_RESPONSE | Immune response | 1005 |
| GO_IMMUNE_SYSTEM_DEVELOPMENT | Immune response | 990 |
| GO_POSITIVE_REGULATION_OF_IMMUNE_RESPONSE | Immune response | 895 |
| REACTOME_CYTOKINE_SIGNALING_IN_IMMUNE_SYSTEM | Immune response | 858 |
| REACTOME_ADAPTIVE_IMMUNE_SYSTEM | Immune response | 818 |
| GO_ACTIVATION_OF_IMMUNE_RESPONSE | Immune response | 720 |
| GO_CELL_ACTIVATION_INVOLVED_IN_IMMUNE_RESPONSE | Immune response | 710 |
| GO_IMMUNE_RESPONSE_REGULATING_SIGNALING_PATHWAY | Immune response | 674 |
| GO_ADAPTIVE_IMMUNE_RESPONSE | Immune response | 663 |
| GO_IMMUNE_RESPONSE_REGULATING_CELL_SURFACE_RECEPTOR_SIGNALING_PATHWAY | Immune response | 500 |
| GO_NEGATIVE_REGULATION_OF_IMMUNE_SYSTEM_PROCESS | Immune response | 481 |
| GO_REGULATION_OF_IMMUNE_EFFECTOR_PROCESS | Immune response | 455 |
| GO_ADAPTIVE_IMMUNE_RESPONSE_BASED_ON_SOMATIC_RECOMBINATION_OF_IMMUNE_RECEPTORS_BUILT_FROM_IMMUNOGLOBULIN_SUPERFAMILY_DOMAINS | Immune response | 352 |
| GO_HUMORAL_IMMUNE_RESPONSE | Immune response | 352 |
| GO_ACTIVATION_OF_INNATE_IMMUNE_RESPONSE | Immune response | 322 |
| GO_PRODUCTION_OF_MOLECULAR_MEDIATOR_OF_IMMUNE_RESPONSE | Immune response | 298 |
| GO_POSITIVE_REGULATION_OF_IMMUNE_EFFECTOR_PROCESS | Immune response | 213 |
| GO_LYMPHOCYTE_ACTIVATION_INVOLVED_IN_IMMUNE_RESPONSE | Immune response | 182 |
| GO_REGULATION_OF_ADAPTIVE_IMMUNE_RESPONSE | Immune response | 160 |
| GO_NEGATIVE_REGULATION_OF_IMMUNE_RESPONSE | Immune response | 157 |
| GO_HUMORAL_IMMUNE_RESPONSE_MEDIATED_BY_CIRCULATING_IMMUNOGLOBULIN | Immune response | 147 |
| GO_REGULATION_OF_PRODUCTION_OF_MOLECULAR_MEDIATOR_OF_IMMUNE_RESPONSE | Immune response | 142 |
| GO_REGULATION_OF_HUMORAL_IMMUNE_RESPONSE | Immune response | 133 |
| GO_IMMUNE_RECEPTOR_ACTIVITY | Immune response | 129 |
| GO_NEGATIVE_REGULATION_OF_IMMUNE_EFFECTOR_PROCESS | Immune response | 121 |
| GO_INNATE_IMMUNE_RESPONSE_ACTIVATING_CELL_SURFACE_RECEPTOR_SIGNALING_PATHWAY | Immune response | 115 |
| GO_T_CELL_ACTIVATION_INVOLVED_IN_IMMUNE_RESPONSE | Immune response | 106 |
| GO_POSITIVE_REGULATION_OF_ADAPTIVE_IMMUNE_RESPONSE | Immune response | 105 |
| GO_CYTOKINE_PRODUCTION_INVOLVED_IN_IMMUNE_RESPONSE | Immune response | 105 |
| GO_POSITIVE_REGULATION_OF_PRODUCTION_OF_MOLECULAR_MEDIATOR_OF_IMMUNE_RESPONSE | Immune response | 97 |
| GO_REGULATION_OF_CYTOKINE_PRODUCTION_INVOLVED_IN_IMMUNE_RESPONSE | Immune response | 86 |
| GALINDO_IMMUNE_RESPONSE_TO_ENTEROTOXIN | Immune response | 80 |
| GO_SOMATIC_DIVERSIFICATION_OF_IMMUNE_RECEPTORS | Immune response | 75 |
| GO_B_CELL_ACTIVATION_INVOLVED_IN_IMMUNE_RESPONSE | Immune response | 74 |
| GO_ANTIMICROBIAL_HUMORAL_IMMUNE_RESPONSE_MEDIATED_BY_ANTIMICROBIAL_PEPTIDE | Immune response | 74 |
| GO_T_CELL_DIFFERENTIATION_INVOLVED_IN_IMMUNE_RESPONSE | Immune response | 68 |
| GOLDRATH_IMMUNE_MEMORY | Immune response | 64 |
| GO_SOMATIC_DIVERSIFICATION_OF_IMMUNE_RECEPTORS_VIA_GERMLINE_RECOMBINATION_WITHIN_A_SINGLE_LOCUS | Immune response | 63 |
| GO_NEGATIVE_REGULATION_OF_INNATE_IMMUNE_RESPONSE | Immune response | 60 |
| GO_IMMUNOGLOBULIN_PRODUCTION_INVOLVED_IN_IMMUNOGLOBULIN_MEDIATED_IMMUNE_RESPONSE | Immune response | 57 |
| GO_POSITIVE_REGULATION_OF_CYTOKINE_PRODUCTION_INVOLVED_IN_IMMUNE_RESPONSE | Immune response | 56 |
| KEGG_AUTOIMMUNE_THYROID_DISEASE | Immune response | 52 |
| KEGG_INTESTINAL_IMMUNE_NETWORK_FOR_IGA_PRODUCTION | Immune response | 48 |
| GO_NEGATIVE_REGULATION_OF_ADAPTIVE_IMMUNE_RESPONSE | Immune response | 46 |
| GO_T_HELPER_1_TYPE_IMMUNE_RESPONSE | Immune response | 42 |
| GO_ORGAN_OR_TISSUE_SPECIFIC_IMMUNE_RESPONSE | Immune response | 39 |
| GO_TYPE_2_IMMUNE_RESPONSE | Immune response | 38 |
| GO_NEGATIVE_REGULATION_OF_PRODUCTION_OF_MOLECULAR_MEDIATOR_OF_IMMUNE_RESPONSE | Immune response | 36 |
| GO_NATURAL_KILLER_CELL_ACTIVATION_INVOLVED_IN_IMMUNE_RESPONSE | Immune response | 32 |
| GO_REGULATION_OF_TYPE_2_IMMUNE_RESPONSE | Immune response | 31 |
| GO_REGULATION_OF_MAST_CELL_ACTIVATION_INVOLVED_IN_IMMUNE_RESPONSE | Immune response | 30 |
| GO_T_HELPER_17_TYPE_IMMUNE_RESPONSE | Immune response | 29 |
| GO_REGULATION_OF_T_HELPER_1_TYPE_IMMUNE_RESPONSE | Immune response | 26 |
| GO_NEGATIVE_REGULATION_OF_CYTOKINE_PRODUCTION_INVOLVED_IN_IMMUNE_RESPONSE | Immune response | 25 |
| GO_INNATE_IMMUNE_RESPONSE_IN_MUCOSA | Immune response | 24 |
| REACTOME_DISEASES_OF_IMMUNE_SYSTEM | Immune response | 24 |
| GO_REGULATION_OF_T_HELPER_17_TYPE_IMMUNE_RESPONSE | Immune response | 21 |
| LIN_TUMOR_ESCAPE_FROM_IMMUNE_ATTACK | Immune response | 20 |
| GO_CYTOKINE_SECRETION_INVOLVED_IN_IMMUNE_RESPONSE | Immune response | 20 |
| GO_POSITIVE_REGULATION_OF_T_HELPER_1_TYPE_IMMUNE_RESPONSE | Immune response | 19 |
| GO_MATURE_B_CELL_DIFFERENTIATION_INVOLVED_IN_IMMUNE_RESPONSE | Immune response | 19 |
| GO_POSITIVE_REGULATION_OF_MYELOID_LEUKOCYTE_CYTOKINE_PRODUCTION_INVOLVED_IN_IMMUNE_RESPONSE | Immune response | 19 |
| GO_POSITIVE_REGULATION_OF_HUMORAL_IMMUNE_RESPONSE | Immune response | 19 |
| GO_SOMATIC_DIVERSIFICATION_OF_IMMUNE_RECEPTORS_VIA_SOMATIC_MUTATION | Immune response | 18 |
| GO_IMMUNE_RESPONSE_TO_TUMOR_CELL | Immune response | 17 |
| GO_POSITIVE_REGULATION_OF_TYPE_2_IMMUNE_RESPONSE | Immune response | 16 |
| REACTOME_STING_MEDIATED_INDUCTION_OF_HOST_IMMUNE_RESPONSES | Immune response | 16 |
| GO_REGULATION_OF_CYTOKINE_SECRETION_INVOLVED_IN_IMMUNE_RESPONSE | Immune response | 16 |
| REACTOME_REGULATION_OF_INNATE_IMMUNE_RESPONSES_TO_CYTOSOLIC_DNA | Immune response | 15 |
| GO_NEGATIVE_REGULATION_OF_TYPE_2_IMMUNE_RESPONSE | Immune response | 14 |
| GO_POSITIVE_REGULATION_OF_MAST_CELL_ACTIVATION_INVOLVED_IN_IMMUNE_RESPONSE | Immune response | 14 |
| GO_MACROPHAGE_ACTIVATION_INVOLVED_IN_IMMUNE_RESPONSE | Immune response | 13 |
| GO_NEGATIVE_REGULATION_OF_HUMORAL_IMMUNE_RESPONSE | Immune response | 12 |
| GO_REGULATION_OF_HUMORAL_IMMUNE_RESPONSE_MEDIATED_BY_CIRCULATING_IMMUNOGLOBULIN | Immune response | 11 |
| GO_POSITIVE_REGULATION_OF_T_HELPER_17_TYPE_IMMUNE_RESPONSE | Immune response | 11 |
| REACTOME_SUMOYLATION_OF_IMMUNE_RESPONSE_PROTEINS | Immune response | 11 |
| GO_NEGATIVE_REGULATION_OF_T_HELPER_17_TYPE_IMMUNE_RESPONSE | Immune response | 10 |
| GO_NATURAL_KILLER_CELL_MEDIATED_IMMUNE_RESPONSE_TO_TUMOR_CELL | Immune response | 9 |
| GO_POSITIVE_REGULATION_OF_CYTOKINE_SECRETION_INVOLVED_IN_IMMUNE_RESPONSE | Immune response | 8 |
| GO_IMMUNE_RESPONSE_INHIBITING_SIGNAL_TRANSDUCTION | Immune response | 8 |
| GO_NEGATIVE_REGULATION_OF_MAST_CELL_ACTIVATION_INVOLVED_IN_IMMUNE_RESPONSE | Immune response | 8 |
| GO_NEGATIVE_REGULATION_OF_T_HELPER_1_TYPE_IMMUNE_RESPONSE | Immune response | 7 |
| GO_POSITIVE_REGULATION_OF_NATURAL_KILLER_CELL_MEDIATED_IMMUNE_RESPONSE_TO_TUMOR_CELL | Immune response | 7 |
| REACTOME_MODULATION_BY_MTB_OF_HOST_IMMUNE_SYSTEM | Immune response | 7 |
| GO_B_CELL_PROLIFERATION_INVOLVED_IN_IMMUNE_RESPONSE | Immune response | 6 |
| GO_NEGATIVE_REGULATION_OF_CYTOKINE_SECRETION_INVOLVED_IN_IMMUNE_RESPONSE | Immune response | 6 |
| GO_IMMUNE_RESPONSE_INHIBITING_CELL_SURFACE_RECEPTOR_SIGNALING_PATHWAY | Immune response | 6 |
| GO_NEGATIVE_REGULATION_OF_HUMORAL_IMMUNE_RESPONSE_MEDIATED_BY_CIRCULATING_IMMUNOGLOBULIN | Immune response | 6 |
| GO_ANTIFUNGAL_INNATE_IMMUNE_RESPONSE | Immune response | 6 |
| REACTOME_RUNX3_REGULATES_IMMUNE_RESPONSE_AND_CELL_MIGRATION | Immune response | 6 |
| GO_TOLERANCE_INDUCTION_DEPENDENT_UPON_IMMUNE_RESPONSE | Immune response | 5 |
| GO_POSITIVE_REGULATION_OF_HUMORAL_IMMUNE_RESPONSE_MEDIATED_BY_CIRCULATING_IMMUNOGLOBULIN | Immune response | 5 |
| GO_REGULATION_OF_NATURAL_KILLER_CELL_DIFFERENTIATION_INVOLVED_IN_IMMUNE_RESPONSE | Immune response | 5 |
| GO_LIPOPOLYSACCHARIDE_IMMUNE_RECEPTOR_ACTIVITY | Immune response | 5 |
| GO_PEPTIDOGLYCAN_IMMUNE_RECEPTOR_ACTIVITY | Immune response | 5 |
| GO_T_CELL_PROLIFERATION_INVOLVED_IN_IMMUNE_RESPONSE | Immune response | 5 |
| REACTOME_METABOLISM_OF_LIPIDS | Cell metabolism | 739 |
| REACTOME_METABOLISM_OF_RNA | Cell metabolism | 672 |
| REACTOME_METABOLISM_OF_AMINO_ACIDS_AND_DERIVATIVES | Cell metabolism | 374 |
| REACTOME_METABOLISM_OF_CARBOHYDRATES | Cell metabolism | 293 |
| REACTOME_DISEASES_OF_METABOLISM | Cell metabolism | 246 |
| REACTOME_PHOSPHOLIPID_METABOLISM | Cell metabolism | 211 |
| HALLMARK_XENOBIOTIC_METABOLISM | Cell metabolism | 200 |
| HALLMARK_HEME_METABOLISM | Cell metabolism | 200 |
| REACTOME_METABOLISM_OF_VITAMINS_AND_COFACTORS | Cell metabolism | 189 |
| REACTOME_FATTY_ACID_METABOLISM | Cell metabolism | 177 |
| KEGG_PURINE_METABOLISM | Cell metabolism | 159 |
| HALLMARK_FATTY_ACID_METABOLISM | Cell metabolism | 158 |
| REACTOME_METABOLISM_OF_STEROIDS | Cell metabolism | 151 |
| REACTOME_GLYCOSAMINOGLYCAN_METABOLISM | Cell metabolism | 124 |
| REACTOME_METABOLISM_OF_WATER_SOLUBLE_VITAMINS_AND_COFACTORS | Cell metabolism | 123 |
| REACTOME_REGULATION_OF_LIPID_METABOLISM_BY_PPARALPHA | Cell metabolism | 119 |
| REACTOME_SELENOAMINO_ACID_METABOLISM | Cell metabolism | 118 |
| HALLMARK_BILE_ACID_METABOLISM | Cell metabolism | 112 |
| REACTOME_INTEGRATION_OF_ENERGY_METABOLISM | Cell metabolism | 108 |
| REACTOME_METABOLISM_OF_NUCLEOTIDES | Cell metabolism | 99 |
| KEGG_PYRIMIDINE_METABOLISM | Cell metabolism | 98 |
| CHEN_LIVER_METABOLISM_QTL_CIS | Cell metabolism | 95 |
| REACTOME_GLUCOSE_METABOLISM | Cell metabolism | 92 |
| REACTOME_PEPTIDE_HORMONE_METABOLISM | Cell metabolism | 90 |
| REACTOME_SPHINGOLIPID_METABOLISM | Cell metabolism | 89 |
| REACTOME_PI_METABOLISM | Cell metabolism | 84 |
| KEGG_GLYCEROPHOSPHOLIPID_METABOLISM | Cell metabolism | 77 |
| KEGG_DRUG_METABOLISM_CYTOCHROME_P450 | Cell metabolism | 72 |
| KEGG_METABOLISM_OF_XENOBIOTICS_BY_CYTOCHROME_P450 | Cell metabolism | 70 |
| KEGG_RETINOL_METABOLISM | Cell metabolism | 64 |
| REACTOME_METABOLISM_OF_POLYAMINES | Cell metabolism | 59 |
| REACTOME_ARACHIDONIC_ACID_METABOLISM | Cell metabolism | 59 |
| KEGG_ARACHIDONIC_ACID_METABOLISM | Cell metabolism | 58 |
| REACTOME_PYRUVATE_METABOLISM_AND_CITRIC_ACID_TCA_CYCLE | Cell metabolism | 55 |
| REACTOME_HEPARAN_SULFATE_HEPARIN_HS_GAG_METABOLISM | Cell metabolism | 55 |
| KEGG_ARGININE_AND_PROLINE_METABOLISM | Cell metabolism | 54 |
| KEGG_INOSITOL_PHOSPHATE_METABOLISM | Cell metabolism | 54 |
| KEGG_STARCH_AND_SUCROSE_METABOLISM | Cell metabolism | 52 |
| KEGG_DRUG_METABOLISM_OTHER_ENZYMES | Cell metabolism | 51 |
| KEGG_GLUTATHIONE_METABOLISM | Cell metabolism | 50 |
| REACTOME_CHONDROITIN_SULFATE_DERMATAN_SULFATE_METABOLISM | Cell metabolism | 50 |
| KEGG_GLYCEROLIPID_METABOLISM | Cell metabolism | 49 |
| REACTOME_INOSITOL_PHOSPHATE_METABOLISM | Cell metabolism | 48 |
| REACTOME_METABOLISM_OF_FAT_SOLUBLE_VITAMINS | Cell metabolism | 48 |
| REACTOME_GLYCOSPHINGOLIPID_METABOLISM | Cell metabolism | 45 |
| KEGG_AMINO_SUGAR_AND_NUCLEOTIDE_SUGAR_METABOLISM | Cell metabolism | 44 |
| REACTOME_BILE_ACID_AND_BILE_SALT_METABOLISM | Cell metabolism | 43 |
| KEGG_FATTY_ACID_METABOLISM | Cell metabolism | 42 |
| KEGG_TYROSINE_METABOLISM | Cell metabolism | 42 |
| KEGG_PORPHYRIN_AND_CHLOROPHYLL_METABOLISM | Cell metabolism | 41 |
| REACTOME_DISEASES_ASSOCIATED_WITH_GLYCOSAMINOGLYCAN_METABOLISM | Cell metabolism | 41 |
| KEGG_TRYPTOPHAN_METABOLISM | Cell metabolism | 40 |
| KEGG_PYRUVATE_METABOLISM | Cell metabolism | 40 |
| KEGG_SPHINGOLIPID_METABOLISM | Cell metabolism | 39 |
| REACTOME_TRIGLYCERIDE_METABOLISM | Cell metabolism | 38 |
| REACTOME_METABOLISM_OF_STEROID_HORMONES | Cell metabolism | 35 |
| KEGG_FRUCTOSE_AND_MANNOSE_METABOLISM | Cell metabolism | 34 |
| KEGG_CYSTEINE_AND_METHIONINE_METABOLISM | Cell metabolism | 34 |
| KEGG_BUTANOATE_METABOLISM | Cell metabolism | 34 |
| REACTOME_KERATAN_SULFATE_KERATIN_METABOLISM | Cell metabolism | 34 |
| REACTOME_DISEASES_OF_CARBOHYDRATE_METABOLISM | Cell metabolism | 34 |
| KEGG_ETHER_LIPID_METABOLISM | Cell metabolism | 33 |
| KEGG_PROPANOATE_METABOLISM | Cell metabolism | 33 |
| REACTOME_SIALIC_ACID_METABOLISM | Cell metabolism | 33 |
| KEGG_ALANINE_ASPARTATE_AND_GLUTAMATE_METABOLISM | Cell metabolism | 32 |
| KEGG_GLYCINE_SERINE_AND_THREONINE_METABOLISM | Cell metabolism | 31 |
| REACTOME_PYRUVATE_METABOLISM | Cell metabolism | 31 |
| REACTOME_NICOTINATE_METABOLISM | Cell metabolism | 31 |
| REACTOME_GLYOXYLATE_METABOLISM_AND_GLYCINE_DEGRADATION | Cell metabolism | 31 |
| REACTOME_SURFACTANT_METABOLISM | Cell metabolism | 30 |
| KEGG_HISTIDINE_METABOLISM | Cell metabolism | 29 |
| KEGG_LINOLEIC_ACID_METABOLISM | Cell metabolism | 29 |
| REACTOME_PEROXISOMAL_LIPID_METABOLISM | Cell metabolism | 29 |
| REACTOME_SULFUR_AMINO_ACID_METABOLISM | Cell metabolism | 28 |
| REACTOME_GLYCOGEN_METABOLISM | Cell metabolism | 27 |
| KEGG_GALACTOSE_METABOLISM | Cell metabolism | 26 |
| KEGG_SELENOAMINO_ACID_METABOLISM | Cell metabolism | 26 |
| REACTOME_METABOLISM_OF_PORPHYRINS | Cell metabolism | 26 |
| KEGG_ASCORBATE_AND_ALDARATE_METABOLISM | Cell metabolism | 25 |
| KEGG_NICOTINATE_AND_NICOTINAMIDE_METABOLISM | Cell metabolism | 24 |
| KEGG_NITROGEN_METABOLISM | Cell metabolism | 23 |
| KEGG_BETA_ALANINE_METABOLISM | Cell metabolism | 22 |
| REACTOME_DEFECTS_IN_VITAMIN_AND_COFACTOR_METABOLISM | Cell metabolism | 22 |
| MOOTHA_GLYCOGEN_METABOLISM | Cell metabolism | 21 |
| REACTOME_COBALAMIN_CBL_VITAMIN_B12_TRANSPORT_AND_METABOLISM | Cell metabolism | 21 |
| KEGG_ALPHA_LINOLENIC_ACID_METABOLISM | Cell metabolism | 19 |
| REACTOME_METABOLISM_OF_COFACTORS | Cell metabolism | 19 |
| KEGG_PHENYLALANINE_METABOLISM | Cell metabolism | 18 |
| REACTOME_METABOLISM_OF_ANGIOTENSINOGEN_TO_ANGIOTENSINS | Cell metabolism | 18 |
| REACTOME_METABOLISM_OF_AMINE_DERIVED_HORMONES | Cell metabolism | 18 |
| REACTOME_VITAMIN_B5_PANTOTHENATE_METABOLISM | Cell metabolism | 17 |
| REACTOME_HYALURONAN_METABOLISM | Cell metabolism | 17 |
| REACTOME_METABOLISM_OF_FOLATE_AND_PTERINES | Cell metabolism | 17 |
| KEGG_GLYOXYLATE_AND_DICARBOXYLATE_METABOLISM | Cell metabolism | 16 |
| KEGG_RIBOFLAVIN_METABOLISM | Cell metabolism | 16 |
| REACTOME_METABOLISM_OF_NITRIC_OXIDE_ENOS_ACTIVATION_AND_REGULATION | Cell metabolism | 15 |
| REACTOME_CARNITINE_METABOLISM | Cell metabolism | 14 |
| REACTOME_DEFECTS_IN_COBALAMIN_B12_METABOLISM | Cell metabolism | 14 |
| REACTOME_GLUTAMATE_AND_GLUTAMINE_METABOLISM | Cell metabolism | 14 |
| KEGG_SULFUR_METABOLISM | Cell metabolism | 13 |
| GO_AMINOACYL_TRNA_METABOLISM_INVOLVED_IN_TRANSLATIONAL_FIDELITY | Cell metabolism | 13 |
| REACTOME_ALPHA_LINOLENIC_OMEGA3_AND_LINOLEIC_OMEGA6_ACID_METABOLISM | Cell metabolism | 13 |
| REACTOME_REGULATION_OF_GLYCOLYSIS_BY_FRUCTOSE_2_6_BISPHOSPHATE_METABOLISM | Cell metabolism | 12 |
| REACTOME_BIOTIN_TRANSPORT_AND_METABOLISM | Cell metabolism | 11 |
| REACTOME_VITAMIN_D_CALCIFEROL_METABOLISM | Cell metabolism | 11 |
| REACTOME_CREATINE_METABOLISM | Cell metabolism | 11 |
| REACTOME_PHENYLALANINE_AND_TYROSINE_METABOLISM | Cell metabolism | 11 |
| REACTOME_ASPARTATE_AND_ASPARAGINE_METABOLISM | Cell metabolism | 11 |
| KEGG_TAURINE_AND_HYPOTAURINE_METABOLISM | Cell metabolism | 10 |
| REACTOME_ABACAVIR_TRANSPORT_AND_METABOLISM | Cell metabolism | 10 |
| REACTOME_DISEASES_ASSOCIATED_WITH_SURFACTANT_METABOLISM | Cell metabolism | 10 |
| REACTOME_KETONE_BODY_METABOLISM | Cell metabolism | 10 |
| REACTOME_VITAMIN_C_ASCORBATE_METABOLISM | Cell metabolism | 8 |
| REACTOME_LINOLEIC_ACID_LA_METABOLISM | Cell metabolism | 8 |
| REACTOME_METABOLISM_OF_INGESTED_SEMET_SEC_MESEC_INTO_H2SE | Cell metabolism | 8 |
| REACTOME_DEFECTS_IN_BIOTIN_BTN_METABOLISM | Cell metabolism | 8 |
| REACTOME_DEFECTIVE_CSF2RB_CAUSES_PULMONARY_SURFACTANT_METABOLISM_DYSFUNCTION_5_SMDP5 | Cell metabolism | 8 |
| REACTOME_VITAMIN_B2_RIBOFLAVIN_METABOLISM | Cell metabolism | 7 |
| REACTOME_FRUCTOSE_METABOLISM | Cell metabolism | 7 |
| REACTOME_PHENYLALANINE_METABOLISM | Cell metabolism | 6 |
| REACTOME_VITAMIN_B1_THIAMIN_METABOLISM | Cell metabolism | 5 |
| REACTOME_ABACAVIR_METABOLISM | Cell metabolism | 5 |

**Supplementary Table 2.** Core pathways of each TME subtypes.

| **Subtypes** | **Core_pathways** |
| --- | --- |
| C1 | FARDIN_HYPOXIA_11 |
| C1 | KEGG_ALANINE_ASPARTATE_AND_GLUTAMATE_METABOLISM |
| C1 | KEGG_TAURINE_AND_HYPOTAURINE_METABOLISM |
| C1 | KEGG_GLYCEROPHOSPHOLIPID_METABOLISM |
| C1 | KEGG_PYRUVATE_METABOLISM |
| C1 | KEGG_PROPANOATE_METABOLISM |
| C1 | KEGG_BUTANOATE_METABOLISM |
| C1 | REACTOME_PHOSPHOLIPID_METABOLISM |
| C1 | REACTOME_ABACAVIR_TRANSPORT_AND_METABOLISM |
| C1 | REACTOME_INTEGRATION_OF_ENERGY_METABOLISM |
| C1 | GO_AMINOACYL_TRNA_METABOLISM_INVOLVED_IN_TRANSLATIONAL_FIDELITY |
| C1 | REACTOME_CARNITINE_METABOLISM |
| C1 | REACTOME_METABOLISM_OF_AMINE_DERIVED_HORMONES |
| C1 | REACTOME_DEFECTS_IN_BIOTIN_BTN_METABOLISM |
| C1 | REACTOME_GLYOXYLATE_METABOLISM_AND_GLYCINE_DEGRADATION |
| C1 | REACTOME_DEFECTIVE_CSF2RB_CAUSES_PULMONARY_SURFACTANT_METABOLISM_DYSFUNCTION_5_SMDP5 |
| C1 | REACTOME_KETONE_BODY_METABOLISM |
| C1 | REACTOME_METABOLISM_OF_STEROIDS |
| C1 | REACTOME_GLUTAMATE_AND_GLUTAMINE_METABOLISM |
| C2 | WINTER_HYPOXIA_DN |
| C2 | GSE22282_HYPOXIA_VS_NORMOXIA_MYELOID_DC_DN |
| C2 | GO_NATURAL_KILLER_CELL_ACTIVATION_INVOLVED_IN_IMMUNE_RESPONSE |
| C2 | GO_B_CELL_PROLIFERATION_INVOLVED_IN_IMMUNE_RESPONSE |
| C2 | GO_NEGATIVE_REGULATION_OF_CYTOKINE_SECRETION_INVOLVED_IN_IMMUNE_RESPONSE |
| C2 | GO_IMMUNE_RESPONSE_INHIBITING_CELL_SURFACE_RECEPTOR_SIGNALING_PATHWAY |
| C2 | GO_REGULATION_OF_NATURAL_KILLER_CELL_DIFFERENTIATION_INVOLVED_IN_IMMUNE_RESPONSE |
| C2 | GO_REGULATION_OF_T_HELPER_17_TYPE_IMMUNE_RESPONSE |
| C2 | GO_LIPOPOLYSACCHARIDE_IMMUNE_RECEPTOR_ACTIVITY |
| C2 | REACTOME_DISEASES_OF_IMMUNE_SYSTEM |
| C3 | BIOCARTA_P53HYPOXIA_PATHWAY |
| C3 | ELVIDGE_HYPOXIA_UP |
| C3 | ELVIDGE_HYPOXIA_BY_DMOG_UP |
| C3 | WEINMANN_ADAPTATION_TO_HYPOXIA_UP |
| C3 | WEINMANN_ADAPTATION_TO_HYPOXIA_DN |
| C3 | MAINA_HYPOXIA_VHL_TARGETS_UP |
| C3 | KONDO_HYPOXIA |
| C3 | GROSS_HYPOXIA_VIA_HIF1A_ONLY |
| C3 | GROSS_HYPOXIA_VIA_ELK3_DN |
| C3 | GROSS_HYPOXIA_VIA_HIF1A_DN |
| C3 | GROSS_HYPOXIA_VIA_ELK3_ONLY_UP |
| C3 | GROSS_HYPOXIA_VIA_ELK3_AND_HIF1A_UP |
| C3 | MANALO_HYPOXIA_UP |
| C3 | MENSE_HYPOXIA_UP |
| C3 | KIM_HYPOXIA |
| C3 | HARRIS_HYPOXIA |
| C3 | LEONARD_HYPOXIA |
| C3 | JIANG_HYPOXIA_NORMAL |
| C3 | WINTER_HYPOXIA_METAGENE |
| C3 | MIZUKAMI_HYPOXIA_UP |
| C3 | QI_HYPOXIA |
| C3 | WACKER_HYPOXIA_TARGETS_OF_VHL |
| C3 | KRIEG_HYPOXIA_VIA_KDM3A |
| C3 | KRIEG_KDM3A_TARGETS_NOT_HYPOXIA |
| C3 | HALLMARK_HYPOXIA |
| C3 | GSE22282_HYPOXIA_VS_NORMOXIA_MYELOID_DC_UP |
| C3 | GO_HYPOXIA_INDUCIBLE_FACTOR_1ALPHA_SIGNALING_PATHWAY |
| C3 | REACTOME_REGULATION_OF_GENE_EXPRESSION_BY_HYPOXIA_INDUCIBLE_FACTOR |
| C3 | KEGG_INTESTINAL_IMMUNE_NETWORK_FOR_IGA_PRODUCTION |
| C3 | REACTOME_INNATE_IMMUNE_SYSTEM |
| C3 | REACTOME_ADAPTIVE_IMMUNE_SYSTEM |
| C3 | REACTOME_CYTOKINE_SIGNALING_IN_IMMUNE_SYSTEM |
| C3 | GOLDRATH_IMMUNE_MEMORY |
| C3 | GALINDO_IMMUNE_RESPONSE_TO_ENTEROTOXIN |
| C3 | GO_POSITIVE_REGULATION_OF_ADAPTIVE_IMMUNE_RESPONSE |
| C3 | GO_NEGATIVE_REGULATION_OF_ADAPTIVE_IMMUNE_RESPONSE |
| C3 | GO_T_CELL_ACTIVATION_INVOLVED_IN_IMMUNE_RESPONSE |
| C3 | GO_REGULATION_OF_ADAPTIVE_IMMUNE_RESPONSE |
| C3 | GO_POSITIVE_REGULATION_OF_CYTOKINE_PRODUCTION_INVOLVED_IN_IMMUNE_RESPONSE |
| C3 | GO_REGULATION_OF_TYPE_2_IMMUNE_RESPONSE |
| C3 | GO_NEGATIVE_REGULATION_OF_INNATE_IMMUNE_RESPONSE |
| C3 | GO_IMMUNE_SYSTEM_DEVELOPMENT |
| C3 | GO_ADAPTIVE_IMMUNE_RESPONSE_BASED_ON_SOMATIC_RECOMBINATION_OF_IMMUNE_RECEPTORS_BUILT_FROM_IMMUNOGLOBULIN_SUPERFAMILY_DOMAINS |
| C3 | GO_REGULATION_OF_IMMUNE_RESPONSE |
| C3 | GO_REGULATION_OF_CYTOKINE_PRODUCTION_INVOLVED_IN_IMMUNE_RESPONSE |
| C3 | GO_CELL_ACTIVATION_INVOLVED_IN_IMMUNE_RESPONSE |
| C3 | GO_NEGATIVE_REGULATION_OF_TYPE_2_IMMUNE_RESPONSE |
| C3 | GO_INNATE_IMMUNE_RESPONSE_ACTIVATING_CELL_SURFACE_RECEPTOR_SIGNALING_PATHWAY |
| C3 | GO_POSITIVE_REGULATION_OF_TYPE_2_IMMUNE_RESPONSE |
| C3 | GO_HUMORAL_IMMUNE_RESPONSE |
| C3 | GO_POSITIVE_REGULATION_OF_IMMUNE_SYSTEM_PROCESS |
| C3 | GO_INNATE_IMMUNE_RESPONSE_IN_MUCOSA |
| C3 | GO_INNATE_IMMUNE_RESPONSE |
| C3 | GO_REGULATION_OF_IMMUNE_SYSTEM_PROCESS |
| C3 | GO_ADAPTIVE_IMMUNE_RESPONSE |
| C3 | GO_NEGATIVE_REGULATION_OF_HUMORAL_IMMUNE_RESPONSE |
| C3 | GO_ACTIVATION_OF_IMMUNE_RESPONSE |
| C3 | GO_IMMUNOGLOBULIN_PRODUCTION_INVOLVED_IN_IMMUNOGLOBULIN_MEDIATED_IMMUNE_RESPONSE |
| C3 | GO_CYTOKINE_PRODUCTION_INVOLVED_IN_IMMUNE_RESPONSE |
| C3 | GO_POSITIVE_REGULATION_OF_PRODUCTION_OF_MOLECULAR_MEDIATOR_OF_IMMUNE_RESPONSE |
| C3 | GO_HUMORAL_IMMUNE_RESPONSE_MEDIATED_BY_CIRCULATING_IMMUNOGLOBULIN |
| C3 | GO_POSITIVE_REGULATION_OF_IMMUNE_EFFECTOR_PROCESS |
| C3 | GO_REGULATION_OF_HUMORAL_IMMUNE_RESPONSE |
| C3 | GO_MATURE_B_CELL_DIFFERENTIATION_INVOLVED_IN_IMMUNE_RESPONSE |
| C3 | GO_POSITIVE_REGULATION_OF_MYELOID_LEUKOCYTE_CYTOKINE_PRODUCTION_INVOLVED_IN_IMMUNE_RESPONSE |
| C3 | GO_POSITIVE_REGULATION_OF_HUMORAL_IMMUNE_RESPONSE |
| C3 | GO_ACTIVATION_OF_INNATE_IMMUNE_RESPONSE |
| C3 | GO_NEGATIVE_REGULATION_OF_IMMUNE_RESPONSE |
| C3 | GO_REGULATION_OF_IMMUNE_EFFECTOR_PROCESS |
| C3 | GO_PRODUCTION_OF_MOLECULAR_MEDIATOR_OF_IMMUNE_RESPONSE |
| C3 | GO_POSITIVE_REGULATION_OF_IMMUNE_RESPONSE |
| C3 | GO_NEGATIVE_REGULATION_OF_IMMUNE_EFFECTOR_PROCESS |
| C3 | GO_LYMPHOCYTE_ACTIVATION_INVOLVED_IN_IMMUNE_RESPONSE |
| C3 | GO_IMMUNE_EFFECTOR_PROCESS |
| C3 | GO_B_CELL_ACTIVATION_INVOLVED_IN_IMMUNE_RESPONSE |
| C3 | GO_REGULATION_OF_PRODUCTION_OF_MOLECULAR_MEDIATOR_OF_IMMUNE_RESPONSE |
| C3 | GO_IMMUNE_RESPONSE_TO_TUMOR_CELL |
| C3 | GO_POSITIVE_REGULATION_OF_CYTOKINE_SECRETION_INVOLVED_IN_IMMUNE_RESPONSE |
| C3 | GO_IMMUNE_RESPONSE_REGULATING_SIGNALING_PATHWAY |
| C3 | GO_NEGATIVE_REGULATION_OF_T_HELPER_1_TYPE_IMMUNE_RESPONSE |
| C3 | GO_NEGATIVE_REGULATION_OF_HUMORAL_IMMUNE_RESPONSE_MEDIATED_BY_CIRCULATING_IMMUNOGLOBULIN |
| C3 | GO_TYPE_2_IMMUNE_RESPONSE |
| C3 | GO_ANTIFUNGAL_INNATE_IMMUNE_RESPONSE |
| C3 | GO_ANTIMICROBIAL_HUMORAL_IMMUNE_RESPONSE_MEDIATED_BY_ANTIMICROBIAL_PEPTIDE |
| C3 | REACTOME_RUNX3_REGULATES_IMMUNE_RESPONSE_AND_CELL_MIGRATION |
| C3 | GO_T_CELL_PROLIFERATION_INVOLVED_IN_IMMUNE_RESPONSE |
| C3 | GO_REGULATION_OF_CYTOKINE_SECRETION_INVOLVED_IN_IMMUNE_RESPONSE |
| C3 | REACTOME_MODULATION_BY_MTB_OF_HOST_IMMUNE_SYSTEM |
| C3 | KEGG_FRUCTOSE_AND_MANNOSE_METABOLISM |
| C3 | KEGG_GALACTOSE_METABOLISM |
| C3 | KEGG_PHENYLALANINE_METABOLISM |
| C3 | KEGG_GLUTATHIONE_METABOLISM |
| C3 | KEGG_STARCH_AND_SUCROSE_METABOLISM |
| C3 | KEGG_AMINO_SUGAR_AND_NUCLEOTIDE_SUGAR_METABOLISM |
| C3 | KEGG_ARACHIDONIC_ACID_METABOLISM |
| C3 | KEGG_NICOTINATE_AND_NICOTINAMIDE_METABOLISM |
| C3 | REACTOME_METABOLISM_OF_VITAMINS_AND_COFACTORS |
| C3 | REACTOME_GLYCOSPHINGOLIPID_METABOLISM |
| C3 | REACTOME_HYALURONAN_METABOLISM |
| C3 | REACTOME_CHONDROITIN_SULFATE_DERMATAN_SULFATE_METABOLISM |
| C3 | REACTOME_KERATAN_SULFATE_KERATIN_METABOLISM |
| C3 | REACTOME_HEPARAN_SULFATE_HEPARIN_HS_GAG_METABOLISM |
| C3 | REACTOME_GLYCOSAMINOGLYCAN_METABOLISM |
| C3 | REACTOME_SPHINGOLIPID_METABOLISM |
| C3 | REACTOME_METABOLISM_OF_CARBOHYDRATES |
| C3 | CHEN_LIVER_METABOLISM_QTL_CIS |
| C3 | MOOTHA_GLYCOGEN_METABOLISM |
| C3 | HALLMARK_XENOBIOTIC_METABOLISM |
| C3 | REACTOME_VITAMIN_D_CALCIFEROL_METABOLISM |
| C3 | REACTOME_NICOTINATE_METABOLISM |
| C3 | REACTOME_METABOLISM_OF_WATER_SOLUBLE_VITAMINS_AND_COFACTORS |
| C3 | REACTOME_METABOLISM_OF_ANGIOTENSINOGEN_TO_ANGIOTENSINS |
| C3 | REACTOME_DISEASES_ASSOCIATED_WITH_GLYCOSAMINOGLYCAN_METABOLISM |
| C3 | REACTOME_DISEASES_OF_CARBOHYDRATE_METABOLISM |
| C3 | REACTOME_DISEASES_OF_METABOLISM |
| C3 | REACTOME_METABOLISM_OF_FAT_SOLUBLE_VITAMINS |
| C3 | REACTOME_GLYCOGEN_METABOLISM |
| C4 | ELVIDGE_HYPOXIA_BY_DMOG_DN |
| C4 | GROSS_HYPOXIA_VIA_ELK3_UP |
| C4 | GROSS_HYPOXIA_VIA_HIF1A_UP |
| C4 | GROSS_HYPOXIA_VIA_ELK3_ONLY_DN |
| C4 | MANALO_HYPOXIA_DN |
| C4 | JIANG_HYPOXIA_CANCER |
| C4 | JIANG_HYPOXIA_VIA_VHL |
| C4 | GSE26023_PHD3_KO_VS_WT_NEUTROPHIL_HYPOXIA_DN |
| C4 | GO_SOMATIC_DIVERSIFICATION_OF_IMMUNE_RECEPTORS_VIA_SOMATIC_MUTATION |
| C4 | KEGG_PURINE_METABOLISM |
| C4 | KEGG_PYRIMIDINE_METABOLISM |
| C4 | KEGG_CYSTEINE_AND_METHIONINE_METABOLISM |
| C4 | KEGG_SELENOAMINO_ACID_METABOLISM |
| C4 | KEGG_GLYOXYLATE_AND_DICARBOXYLATE_METABOLISM |
| C4 | REACTOME_METABOLISM_OF_AMINO_ACIDS_AND_DERIVATIVES |
| C4 | REACTOME_METABOLISM_OF_RNA |
| C4 | REACTOME_GLUCOSE_METABOLISM |
| C4 | REACTOME_METABOLISM_OF_INGESTED_SEMET_SEC_MESEC_INTO_H2SE |
| C4 | REACTOME_SELENOAMINO_ACID_METABOLISM |
| C4 | REACTOME_METABOLISM_OF_COFACTORS |

**Supplementary Table 3.** Univariate and multivariate analysis of OS in CGGA dataset1

| **Variables** | **Univariate analysis** | | **Multivariate analysis** | |
| --- | --- | --- | --- | --- |
|  | **HR (95% CI)** | **p value** | **HR (95% CI)** | **p value** |
| **Risk score** | 2.862 (2.274-3.602) | < 0.001 | 1.796 (1.224-2.593) | 0.002 |
| **Age at Diagnosis** | 1.028 (1.019-1.038) | < 0.001 | 1.006 (0.997-1.016) | 0.197 |
| **Gender** | 1.024 (0.821-1.277) | 0.836 | - | - |
| **WHO Grade** | 2.924 (2.478-3.449) | < 0.001 | 2.189 (1.753-2.734) | < 0.001 |
| **TCGA Subtype** | 1.561 (1.398-1.774) | < 0.001 | 1.088 (0.938-1.263) | 0.265 |
| **IDH mutation status** | 0.286 (0.226-0.361) | < 0.001 | 0.751 (0.540-1.045) | 0.089 |
| **MGMT methylation** | 0.807 (0.634-1.028) | 0.083 | - | - |
| **1p19q co-deletion** | 0.270 (0.187-0.391) | < 0.001 | 0.543 (0.351-0.839) | 0.006 |
| **Radiotherapy** | 1.490 (1.069-2.078) | 0.019 | 0.923 (0.617-1.380) | 0.695 |
| **Chemotherapy** | 1.528 (1.144-2.043) | 0.004 | 0.804 (0.558-1.158) | 0.241 |

**Supplementary Table 4.** Univariate and multivariate analysis of OS in CGGA dataset2

| **Variables** | **Univariate analysis** | | **Multivariate analysis** | |
| --- | --- | --- | --- | --- |
|  | **HR (95% CI)** | **p value** | **HR (95% CI)** | **p value** |
| **Risk score** | 4.697 (3.541-6.230) | < 0.001 | 3.990 (2.058-7.733) | < 0.001 |
| **Age at Diagnosis** | 1.038 (1.023-1.054) | < 0.001 | 0.995 (0.947-1.016) | 0.622 |
| **Gender** | 1.181 (0.837-1.666) | 0.345 | - | - |
| **WHO Grade** | 3.477 (2.716-4.452) | < 0.001 | 1.825 (1.222-2.724) | 0.030 |
| **TCGA Subtype** | 1.815 (1.557-2.114) | < 0.001 | 0.973 (0.758-1.248) | 0.827 |
| **IDH mutation status** | 0.228 (0.158-0.329) | < 0.001 | 1.565 (0.701-3.496) | 0.275 |
| **MGMT methylation** | 0.528 (0.374-0.450) | < 0.001 | 0.731 (0.467-1.143) | 0.170 |
| **1p19q co-deletion** | 0.134 (0.049-0.363) | < 0.001 | 0.592 (0.206-1.701) | 0.330 |
| **Radiotherapy** | 0.429 (0.296-0.622) | < 0.001 | 0.404 (0.261-0.626) | < 0.001 |
| **Chemotherapy** | 1.378 (0.963-1.971) | 0.079 | - | - |
